# The transcription factor Zt107320 affects the dimorphic switch, growth and virulence of the fungal wheat pathogen *Zymoseptoria tritici*

**DOI:** 10.1111/mpp.12886

**Published:** 2019-11-08

**Authors:** Michael Habig, Sharon Marie Bahena‐Garrido, Friederike Barkmann, Janine Haueisen, Eva Holtgrewe Stukenbrock

**Affiliations:** ^1^ Environmental Genomics Christian‐Albrechts University of Kiel Kiel Germany; ^2^ Max Planck Institute for Evolutionary Biology Plön Germany; ^3^ Present address: National Research Institute of Brewing 3‐7‐1 Kagamiyama Higashi‐Hiroshima 739‐0046 Japan

**Keywords:** fungal dimorphism, gene expression, incompatible host, pycnidia formation, Septoria leaf blotch

## Abstract

*Zymoseptoria tritici* is a filamentous fungus causing Septoria tritici blotch in wheat. The pathogen has a narrow host range and infections of grasses other than susceptible wheat are blocked early after stomatal penetration. During these abortive infections, the fungus shows a markedly different gene expression pattern. However, the underlying mechanisms causing differential gene expression during host and non‐host interactions are largely unknown, but likely include transcriptional regulators responsible for the onset of an infection programme in compatible hosts. MoCOD1, a member of the fungal Zn(II)_2_Cys_6_ transcription factor family, has been shown to directly affect pathogenicity in the rice blast pathogen *Magnaporthe oryzae*. Here, we analyse the role of the putative transcription factor Zt107320, a homologue of MoCOD1, during infection of compatible and incompatible hosts by *Z. tritici*. We show for the first time that *Zt107320* is differentially expressed in host versus non‐host infections and that lower expression corresponds to an incompatible infection of non‐hosts. Applying reverse genetics approaches, we further show that Zt107320 regulates the dimorphic switch as well as the growth rate of *Z. tritici* and affects fungal cell wall composition *in vitro*. Moreover, ∆*Zt107320* mutants showed reduced virulence during compatible infections of wheat. We conclude that Zt107320 directly influences pathogen fitness and propose that Zt107320 is involved in the regulation of growth processes and pathogenicity during infection.

## Introduction

The fungus *Zymoseptoria tritici* (synonym *Mycosphaerella graminicola*) infects wheat and causes the disease Septoria tritici blotch. The pathogen is found worldwide where wheat is grown and can cause severe reduction in yield (Fones and Gurr, 2015). During infection, the fungus enters the leaf through stomata and establishes a hyphal network in the mesophyll. It propagates without causing visual symptoms for 7–14 days before inducing necrosis and producing asexual fructifications, pycnidia, in which pycnidiospores are produced to be dispersed via leaf–leaf contact or rain‐splash to neighbouring leaves (Brading *et al.*, 2002; Kema *et al*. 1996b; Ponomarenko *et al*., 2011). *Zymoseptoria tritici* has a heterothallic mating system and meiosis leads to the production of wind‐borne ascospores that are considered to be the main primary inoculum in wheat fields (Kema *et al*., 1996a; Morais *et al.*, 2016; Ponomarenko *et al*., 2011; Suffert *et al.*, 2011). Under experimental conditions, the fungus has a narrow host range infecting wheat and shows abortive infections on closely related non‐host grass species like *Triticum monococcum* (Jing *et al.*, 2008) and *Brachypodium distachyon* (Kellner *et al.*, 2014; O'Driscoll *et al*., 2015). However, the underlying determinants of host specialization and host specificity of *Z. tritici* are largely unknown.

A previous study comparing the expression profiles of *Z. tritici* between early infection (4 days post‐infection) of the compatible host *Triticum aestivum* and the non‐host *B. distachyon* revealed 289 genes that were similarly expressed in the two hosts, but differentially expressed compared to growth in axenic culture (Kellner *et al.*, 2014). These genes are likely crucial for *Z. tritici* during stomatal penetration, which occurs in the same way in both hosts. However, 40 genes showed differential expression between host and non‐host infections (Kellner *et al.*, 2014) and are possibly involved in the discrimination of compatible and non‐compatible host–pathogen interactions. The signalling and regulatory networks responsible for these differential expression patterns are unknown, however. One of the differentially expressed genes encodes the putative transcription factor Zt107320. Expression of *Zt107320* was significantly increased during infection of *T. aestivum* compared to the early infection of *B. distachyon* (Kellner *et al.*, 2014), suggesting a host‐dependent regulation of the gene.

*Zt107320* encodes a putative transcription factor belonging to the Zn(II)_2_Cys_6_ family. This gene family of transcription factors is exclusive to fungi (MacPherson *et al.*, 2006; Pan and Coleman, 1990) and many members play an important role in the regulation of fungal physiology. For example, Zn(II)_2_Cys_6_ transcription factors in *Magnaporthe oryzae*, *Fusarium oxysporum*, *Leptosphaeria maculans, Parastagonospora nodorum* and *Pyrenophora tritici‐repentis* are involved in the regulation of fungal growth and pathogenicity (Fox *et al.*, 2008; Galhano *et al.*, 2017; Imazaki *et al.*, 2007; Lu *et al.*, 2014; Rybak *et al.*, 2017). Interestingly, the homologue of *Zt107320* in the rice blast pathogen *M. oryzae*, MoCOD1, was shown to affect conidiation and pathogenicity (Chung *et al.*, 2013). *MoCOD1* was found to be up‐regulated during conidiation and appressorium formation at 72 h post‐infection. Furthermore, the deletion mutant ∆*MoCOD1* showed defects in conidial germination and appressorium formation. *In planta*, the mutant ∆*MoCOD1* was attenuated in extending growth from the first‐invaded cells and caused markedly reduced symptoms when compared to the wild type (Chung *et al.*, 2013).

Transcription factors, in general, regulate expression by integrating various signalling pathways and represent interesting targets for dissecting causes and mechanisms of pathogenicity and host specificity. In *M. oryzae,* a systemic approach was applied to characterize all 104 members of the Zn(II)_2_Cys_6_ family of transcription factors. Of these, 61 were shown to be involved in fungal development and pathogenicity (Lu *et al.*, 2014). Similarly, in the head blight‐causing fungus *Fusarium graminearum* 26% of 657 tested transcription factors showed a phenotypic effect during mycelial growth, conidia production and toxin production (Son *et al.*, 2011). In summary, a number of transcription factors, which play important roles in host infection and pathogenesis, have been identified for several important crop pathogens (Chen *et al.*, 2017; Okmen *et al.*, 2014; Xiong *et al.*, 2015; Zhang *et al.*, 2018; Zhuang *et al.*, 2016). In *Z. tritici*, however, only a few regulatory genes have been characterized. Recently, two transcription factors, ZtWor1 (Mirzadi Gohari *et al.*, 2014) and ZtVf1 (Mohammadi *et al.*, 2017), were shown to be important regulators of development and virulence of *Z. tritici* during compatible infections of wheat, highlighting how transcription factors can be used to identify and dissect aspects of pathogenicity. ZtWor1 has been functionally characterized and appears to be involved in the cAMP‐dependent pathway, up‐regulated during the initiation of colonization and involved in regulating effector genes (Mirzadi Gohari *et al.*, 2014). ZtVf1, a transcription factor belonging to the C_2_‐H_2_ subfamily, is required for virulence and its deletion leads to lower pycnidia density within lesions. Decreased virulence appears to be due to a reduced penetration frequency and impaired pycnidia development (Mohammadi *et al.*, 2017). Further regulatory elements that affect pathogenicity in *Z. tritici* encompass several members of mitogen‐activated protein kinase pathways and G‐protein subunits [reviewed in (Rudd, 2015)].

Based on the close homology of *Zt107320* to *MoCOD1* and its significantly different expression profile during host and non‐host infections, we hypothesized that Zt107320 plays an important role in *Z. tritici* during early wheat infection. We show here that Zt107320 affects virulence of *Z. tritici* during compatible infections and regulates the dimorphic switch as well as the growth rate and cell wall properties of this important fungal plant pathogen.

## Results

### Phylogenetic analysis of Zt107320

**Figure 1:**
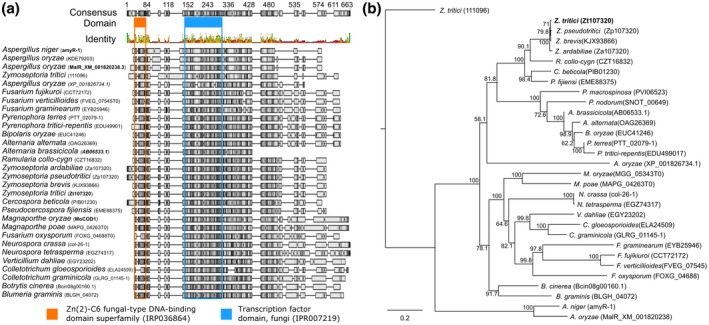
Homology of Zt107320 among fungal transcription factors. (a) MUSCLE alignment (Edgar, 2004) of sequences homologous to Zt107320 that indicates regions according to their identity with the consensus sequence. The localizations of two functional domains, identified using InterProScan, in the consensus sequence are highlighted in orange and blue. (b) Phylogenetic tree based on 30 sequences showing the highest similarity with Zt107320 on the protein level, including the orthologues in the sister species *Zymoseptoria pseudotritici* and *Z. ardabiliae.* The phylogenetic tree was constructed using MUSCLE alignments and neighbour‐joining analysis with 1000 bootstrapping iterations. Shown is the consensus tree. Support of nodes by percentage of bootstrapping iterations is indicated.

In order to determine the distribution of homologues of Zt107320 we performed a similarity search on the protein level among fungal sequences. A total of 30 sequences were retrieved, which included orthologous sequences as well as the non‐orthologous Zt111096, which shows the highest similarity to Zt107320 in *Z. tritici*, and the well‐described transcription factor AmyR and MalR in *Aspergillus niger* and *Aspergillus oryzae*, respectively. These sequences were aligned and a phylogenetic analysis was conducted (Fig. 1a,b). Among these highly conserved proteins two protein domains are shared: Zn(2)‐C6 fungal‐type DNA‐binding domain superfamily (IRP036864) and a transcription factor domain (IRP007219) supporting a functional role of the putative transcription factors (see Fig. 1a). The retrieved sequences contained several proteins that have already been functionally dissected: the *M. oryzae* orthologue MoCod1, the *Alternaria brassicola* orthologue AbPf2 (AB06533.1), the *A. niger* homologue AmyR and the *A. oryzae* homologue MalR. Deletion of *AbPf2* resulted in nonpathogenic strains (Cho *et al.*, 2013). In the wild type, expression of *AbPf2* decreased after initial colonization of host tissues and the authors concluded that AbPf2 regulates pathogenesis (Cho *et al.*, 2013). AmyR in *A. niger* is considered to be a regulator of starch degradation and to regulate the expression of enzymes involved in polysaccharide degradation (Suzuki *et al.*, 2015; vanKuyk *et al.*, 2012; Zhang *et al.*, 2016). MalR in *A. oryzae* is considered to regulate the expression of the maltose‐utilizing cluster genes (Suzuki *et al.*, 2015). Constructing a phylogenetic tree revealed a monophyletic origin of Zt107320 with 27 of the 30 homologues, whereas AmyR, MalR and Zt111096 are non‐monophyletic in regard to Zt107320.

### Infections of *B. distachyon* by *Z. tritici* are blocked in the substomatal cavities during incompatible interaction coinciding with reduced expression of *Zt107320*

**Figure 2:**
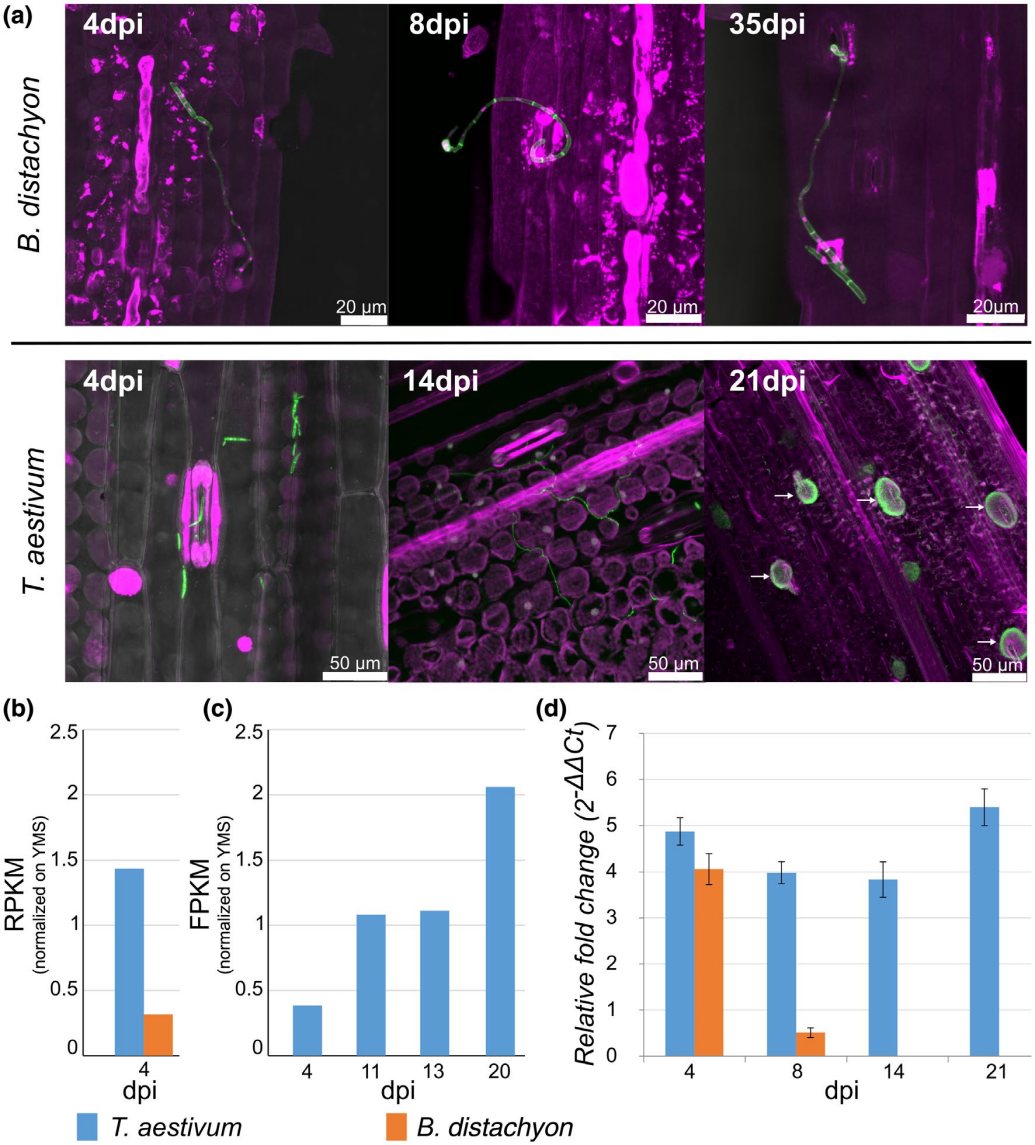
*Zymoseptoria tritici* infection hyphae are blocked in the substomatal cavities of *Brachypodium distachyon,* which coincides with reduced expression of *Zt107320*. (a) Micrographs showing *Z. tritici* cells and emerging hyphae during penetration of *B. distachyon* stomata and infection of *Triticum aestivum*. On *T. aestivum*, spores of *Z. tritici* germinated and hyphae penetrated the stomata at 4 days post‐inoculation (dpi). Intercellular mesophyll colonization of *Z. tritici* was observed until 14 dpi, resulting in the formation of pycnidia (indicated by arrows) at 21 dpi. In contrast, *Z. tritici* spores germinated on *B. distachyon* and hyphae penetrated stomata, but further growth was halted below the stomata with no subsequent leaf colonization (8–35 dpi). Maximum projections of confocal image *z*‐stacks are shown. Nuclei and grass cells displayed in purple and fungal hyphae or septae, respectively, in green. (b) Transcription of *Zt107320* in compatible (*T. aestivum)* and incompatible (*B. distachyon*) hosts at 4 dpi normalized to the expression in YMS medium. Data from Kellner *et al*. (2014). (c) Transcription of *Zt107320* in *T. aestivum* at indicated time points during *in planta* infection normalized to expression of the gene during growth in YMS medium. Data from Haueisen *et al*. (2019). (d) *In planta* expression of *Zt107320* is displayed relative to expression during axenic growth. Values are normalized to the expression of the gene encoding the housekeeping protein GAPDH. Error bars indicate the standard error of the mean (SEM) of three independent biological replicates per sample.

To study compatible and incompatible infections of *Z. tritici* in more detail, we inoculated leaves of 12–14‐day‐old seedlings of *T. aestivum* (cultivar Obelisk) and *B. distachyon* (ecotype Bd21), and analysed the infection development by confocal microscopy. Fungal cells germinated upon contact with the leaf surface and developed infection hyphae. In contrast to previous observations (O'Driscoll *et al*., 2015), we found that *Z. tritici* infection hyphae entered into open *B. distachyon* stomata at 4 days post‐inoculation (dpi). However, further infection development of *Z. tritici* was blocked in the substomatal cavities of *B. distachyon* leaves (Fig. 2a), similar to phenotypes previously observed in incompatible interactions with einkorn wheat (Jing *et al.*, 2008). Consequently, *Z. tritici* hyphae did not colonize the mesophyll tissue of *B. distachyon*, no necrotic lesions developed and no asexual pycnidia formed. Interestingly, fungal growth was completely halted and no further growth was observed, even when leaves were examined 5 weeks post‐inoculation (Fig. 2a). In contrast, *Z. tritici* successfully infected and colonized the mesophyll tissue of the susceptible wheat cultivar Obelisk during different infection stages, as already described in detail (Haueisen *et al.*, 2019). Penetration of leaf stomata at 4 dpi was followed by the establishment of a hyphal network in the mesophyll tissue until 7–11 dpi and subsequently the switch to necrotrophy when biomass increased substantially, resulting in the formation of pycnidia (Fig. 2a).

Previously, comparative transcriptome analyses during *Z. tritici* infection of the host *T. aestivum* and the non‐host *B. distachyon* identified 40 differentially expressed genes at 4 dpi (Kellner *et al.*, 2014) (Fig. 2b). During the course of the infection of a compatible host, a separate study based on transcriptome data showed that the expression of *Zt107320* increases over time, with the highest expression at 20 dpi (Haueisen *et al.*, 2019). This expression pattern is consistent with a putative role of Zt107320 in growth regulation (Fig. 2c). Building on this expression analysis, we focused on the putative transcription factor Zt107320 and validated the expression kinetics of *Zt107320* by RT‐qPCR. We confirmed differential expression at 8 dpi (Fig. 2d). Expression in the non‐host *B. distachyon* was greatly reduced at 8 dpi compared to expression in axenic cultures, whereas the expression of *Zt107320* during a compatible infection of the host *T. aestivum* was strongly up‐regulated during the course of the infection until 21 dpi.

### Zt107320 is located with the nucleus during yeast‐like growth

**Figure 3:**
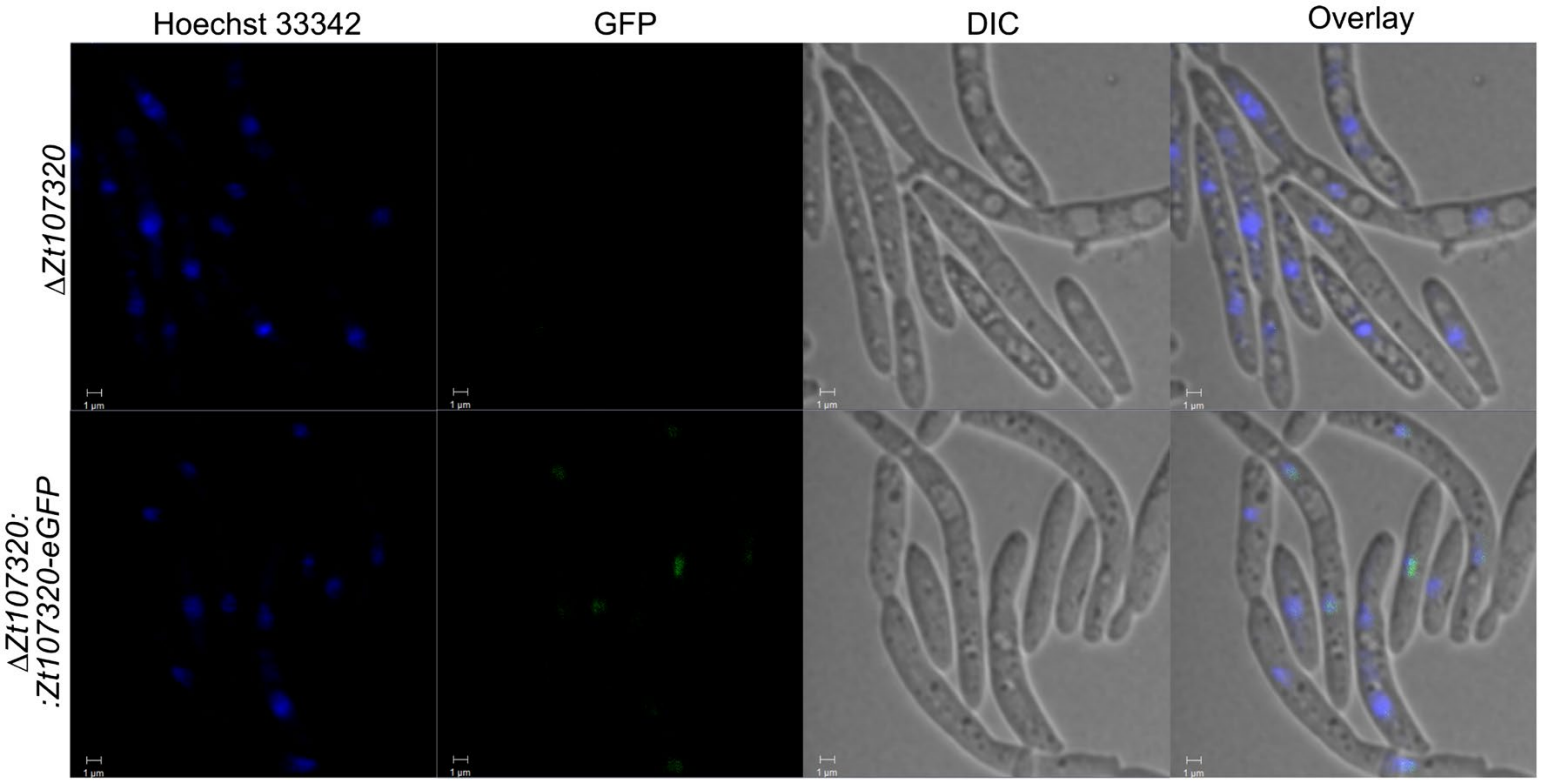
Localization of the Zt107320‐eGFP fusion protein detected by fluorescence microscopy. Nuclei were counterstained using the DNA‐specific dye Hoechst 33342. eGFP fluorescence co‐localized with the Hoechst 33342 signal indicating nuclear localization of the Zt107320‐eGFP fusion protein. eGFP fluorescence was restricted to the nuclei. DIC, differential interference contrast. Scale bars = 1 µm.

The localization of Zt107320 within fungal cells was analysed using complementation strains that expressed a Zt107320‐eGFP fusion protein regulated by the native promoter. During yeast‐like growth on YMS medium, the green fluorescent fusion protein (GFP‐fusion) appeared to be located in the nucleus or in its immediate vicinity (see Fig. 3). This localization is similar to the reported nuclear localization of the homologue AbPf2 (Cho *et al.*, 2013). The nuclear localization of the Zt107320‐eGFP fusion protein, however, was restricted to a fraction of the cells, which could indicate that the protein is also located in the cytoplasm. The observed localization of the Zt107320‐eGFP fusion protein is in concordance with the predicted nuclear localization of Zt107320 using the program WoLFPsort, which implements an algorithm for prediction of subcellular locations of proteins based on sequence composition and content (Horton *et al.*, 2007) and therefore could support the putative functional role of Zt107320 as a transcription factor.

### Zt107320 regulates growth and affects cell wall properties

**Figure 4:**
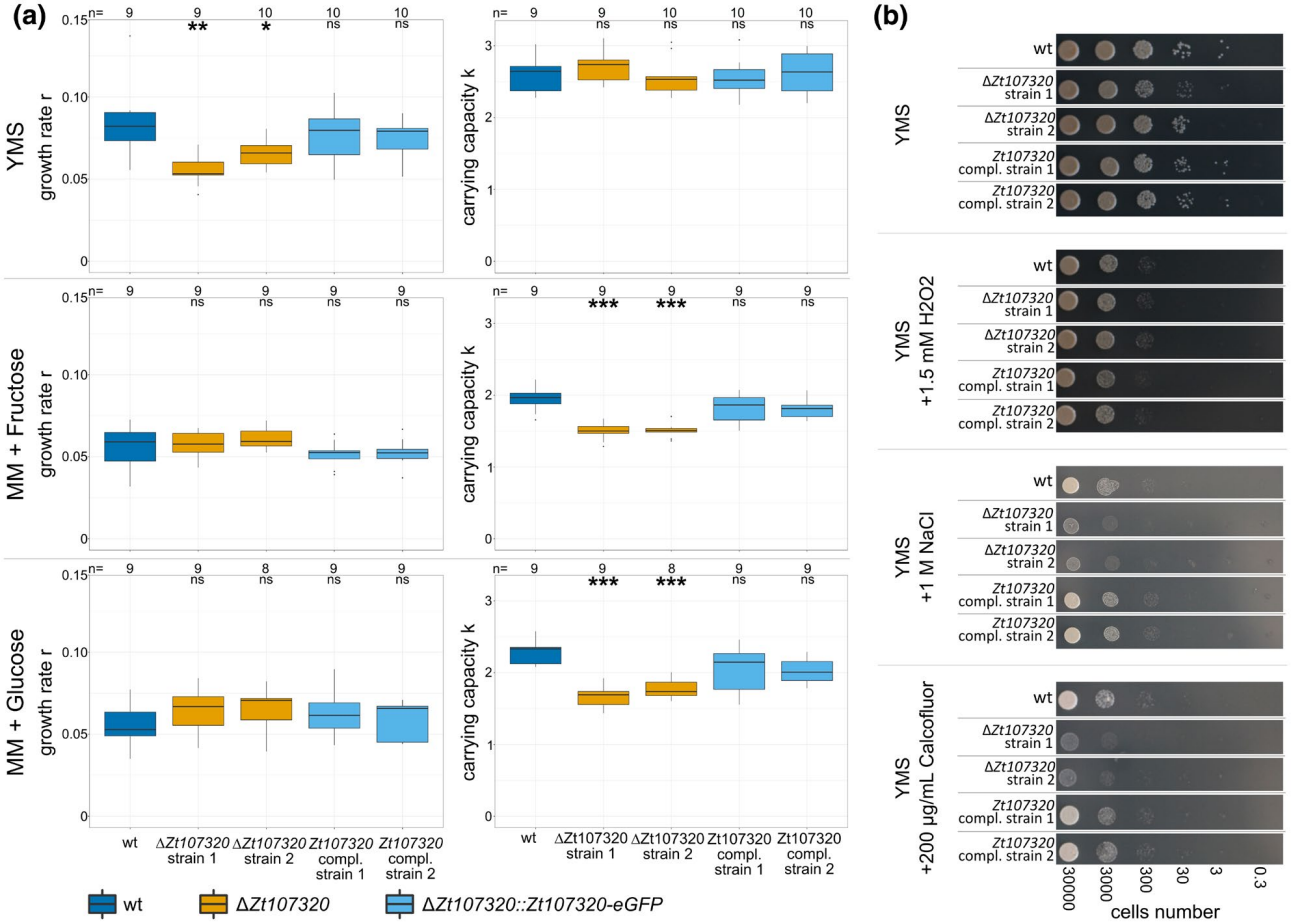
Zt107320 regulates growth and affects cell wall properties of *Zymoseptoria tritici*. (a) Maximum growth rate *r* and carrying capacity *k* measured during fungal growth in liquid culture, in rich complex YMS liquid medium and in liquid minimal medium MM containing fructose and glucose. Statistical significance inferred through an ANOVA and subsequent post hoc Tukey’s HSD comparing the deletion and complementation strains to the wild type is indicated as *P < 0.05, **P < 0.005, ***P < 0.0005. (b) *In vitro* growth of the wild type (wt), two deletion strains (∆*Zt107320*) and two complementation strains (∆*Zt107320*::*Zt107320*‐eGFP) on YMS medium including the supplemented compounds to assess the role of osmotic stress (1 M NaCl), reactive oxygen species (1.5 mM H_2_O_2_) and cell wall stressors (200 µg/mL Calcofluor).

Based on the coinciding halted growth and development and down‐regulation of *Zt107320* in incompatible infections, we next asked whether Zt107320 is involved in the regulation of growth of *Z. tritici in vitro*. We determined the maximum growth rate *r* and the carrying capacity *k* of *Z. tritici* in liquid cultures using rich complex medium (YMS) and chemically defined minimal medium (MM) containing different carbohydrates. Growth was determined by OD_600_ measurements and data fitted assuming a logistic growth curve model. In YMS the two independently generated *Zt107320* deletion mutants achieved a significantly lower maximum growth rate *r* compared to the wild type (median of 65% and 80% for strains 1 and 2, respectively), whereas growth rates were restored to the level of the wild type strain in the two complementation strains (median of 95% and 100%) (Fig. 4a). However, the maximum cell density, which equates to the carrying capacity *k*, was not different from the wild type for the two deletion strains (median of 104% and 96%) or the two complementation strains (median of 95% and 100%). Hence Zt107320 appears to affect the growth rate but not the carrying capacity in rich complex medium. In order to assess the effect of Zt107320 on the growth in conditions that promote hyphal growth and are therefore similar to the growth *in planta* we additionally used MM that included different carbohydrates as energy and carbon sources. Interestingly, in MM the maximal growth rate *r* was not significantly different from the wild type for two deletion strains (median for all MM: 102% and 107% of wild type) and the two complementation strains (median for all MM: 103% and 93% of wild type). However, the carrying capacity was lower for the two deletion strains (median for all MM: 79% and 83%) whereas the complementation strains reached the carrying capacity of the wild type (median for all MM 92% and 94%). The carrying capacity *k* for ∆*Zt107320* was significantly reduced in MM that included fructose, glucose, sucrose and xylose, which are the carbohydrates that showed high carrying capacities (Figs 4a and S1). We thereby confirmed the relevance of Zt107320 as a putative regulator for growth that affects growth rates and carrying capacities. The effect on the carrying capacity but not growth rate in MM could indicate that Zt107320 affects the efficiency with which resources are utilized in MM and thereby affects growth during conditions when resources are limited. In contrast, in rich complex medium a higher overall growth rate can be achieved, with Zt107320 affecting the maximum growth rate but not the carrying capacity.

The effect of *Zt107320* deletion was not restricted to the growth rate but also included cell wall properties. Compared to the wild type, we observed reduced growth *in vitro* when challenging the deletion mutants with high osmotic stresses (0.5 M and 1 M NaCl; 1 M, 1.5 M and 2 M sorbitol) as well as with cell wall stress agents (300 µg/mL and 500 µg/mL Congo red; 200 µg/mL Calcofluor). Again, the wild type phenotype was restored in both complementation strains (Figs 4b and S2). Interestingly, temperature stress (28 °C) did not affect the wild type and the deletion mutants differently, as well as exposure to H_2_O_2_ (1.5 mM and 2 mM). This indicates a specific effect of Zt107320 on the cell wall properties but not on the ability of the fungus to counteract reactive oxygen species, which are produced by the plant during activation of immune responses (Jones and Dangl, 2006).

**Figure 5:**
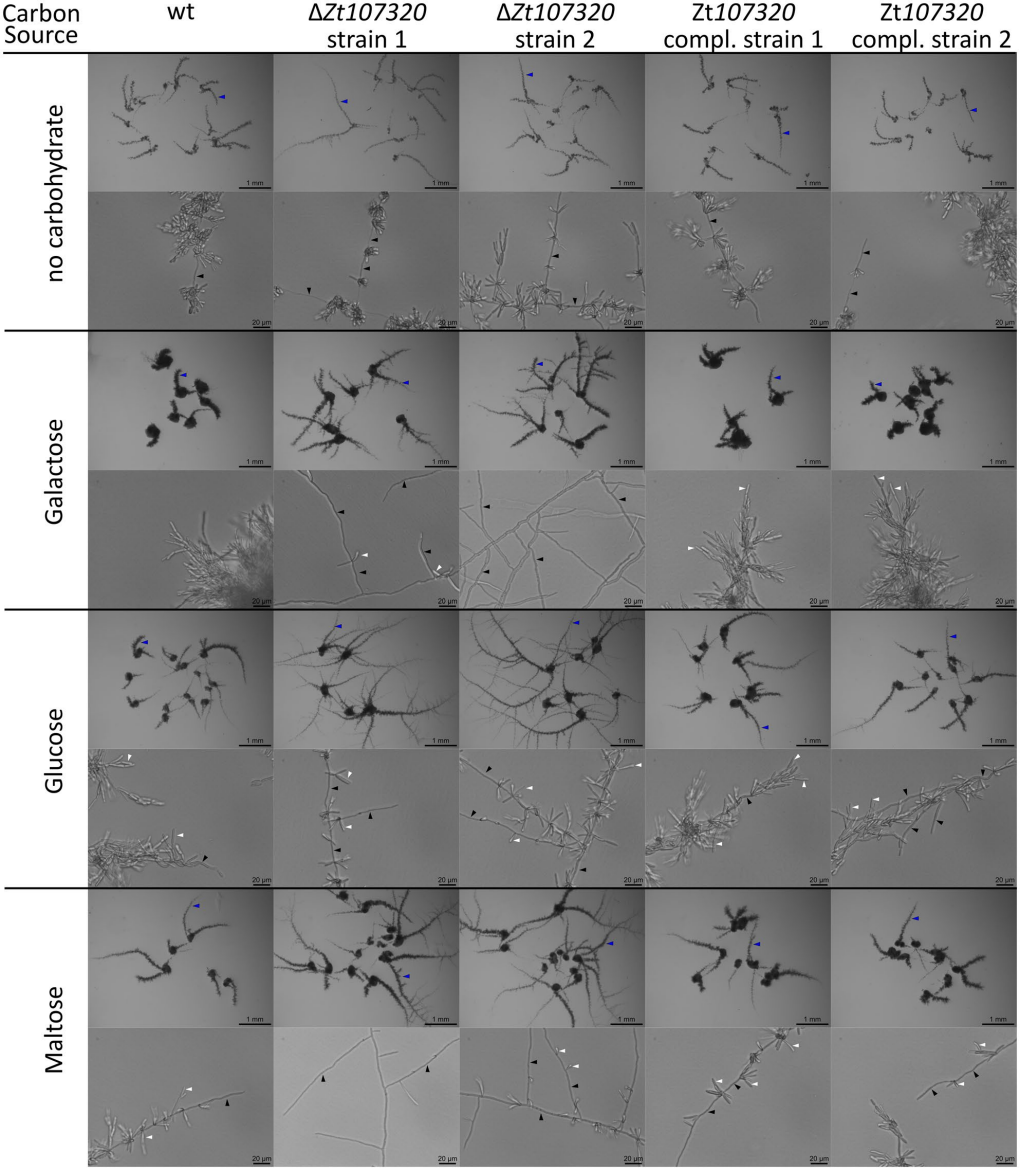
Zt107320 is involved in the regulation of the dimorphic switch of *Zymoseptoria* *tritici*. Pictures showing morphologies of colonies originating from single cells after 14 days of growth at 18 °C on minimal medium (MM) containing the indicated carbohydrates as carbon sources. Upper row for each carbon source: micrographs of colony morphologies. The two independent ∆*Zt107320* strains showed an increase in number and degree of hyphal‐like protrusions (blue arrow marks one example for each condition) from colonies compared to the wild type, while in the two complementation strains the wild type colony morphology was restored. Lower row for each carbon source: micrographs showing cell morphologies in hyphal‐like protrusions from colonies. Under all conditions hyphae (examples are indicated by black arrows) and cells with yeast‐like morphologies (examples are indicated by white arrows) were present. The two ∆*Zt107320* strains showed an increase in the hyphal growth compared to cells with yeast‐like morphologies. Branching of hyphae resulting in hyphal growth was increased in the two ∆*Zt107320* strains whereas in the wild type (wt) and the two complementation strains branching from hyphae produced cells with yeast‐like morphologies (see also Figs S3 and S4).

The dimorphic switch from yeast‐like to hyphal growth is considered to be central for pathogenicity during the early stages of infection (Kema *et al*. 1996b; Motteram *et al.*, 2011; Yemelin *et al.*, 2017). We therefore next asked whether Zt107320 is involved in regulating this morphological switch of *Z. tritici.* To promote hyphal growth we used solid minimal medium and supplied different carbon sources in order to compare carbon utilization and growth between the mutants and wild type. Initially, after 24 h of incubation on the solid media, no differences between the wild type, deletion strains and complementation strains were apparent (data not shown). However, after 14 days of incubation on the respective medium a prominent effect of medium composition and the deletion of *Zt107320* on the growth pattern became apparent. The wild type *Z. tritici* strain showed markedly increased yeast‐like growth in the presence of all tested carbohydrates (Figs 5, S3 and S4). Interestingly, xylose or fructose as sole carbon source led to predominant hyphal growth in the wild type strain, which contrasts with the mainly yeast‐like growth observed in the presence of all other tested carbohydrates. The deletion of *Zt107320* affected fungal growth morphology on all tested carbon sources. Compared to the wild type and the complementation strains, we observed increased hyphal growth for the Δ*Zt107320* strains on all carbon sources after 14 days of incubation (Figs 5, S3 and S4). Microscopic analysis showed the presence of cells with hyphal morphology and yeast‐like morphology in all conditions for the wild type strains, *Zt107320* deletion and complementation strains. Yet, more hyphal growth—and in particular more branching from hyphae that resulted again in hyphal growth—was observed when *Zt107320* was deleted. In contrast, less hyphal growth was observed for the wild type strain or complementation strains and branching of hyphal cells that resulted again in hyphal cells was only observed for these strains in the presence of xylose and fructose but no other carbohydrate (Figs 5, S3 and S4). For fructose and xylose a further increase in hyphal growth and branching compared to the wild type was observed in the two ∆*Zt107320* deletion strains (Fig. S4). Under all conditions the wild type phenotype was restored in both complementation strains, confirming that *Zt107320* plays a role in the regulation of growth in *Z. tritici*.

### Zt107320 affects the ability of *Z. tritici* to produce pycnidia

**Figure 6:**
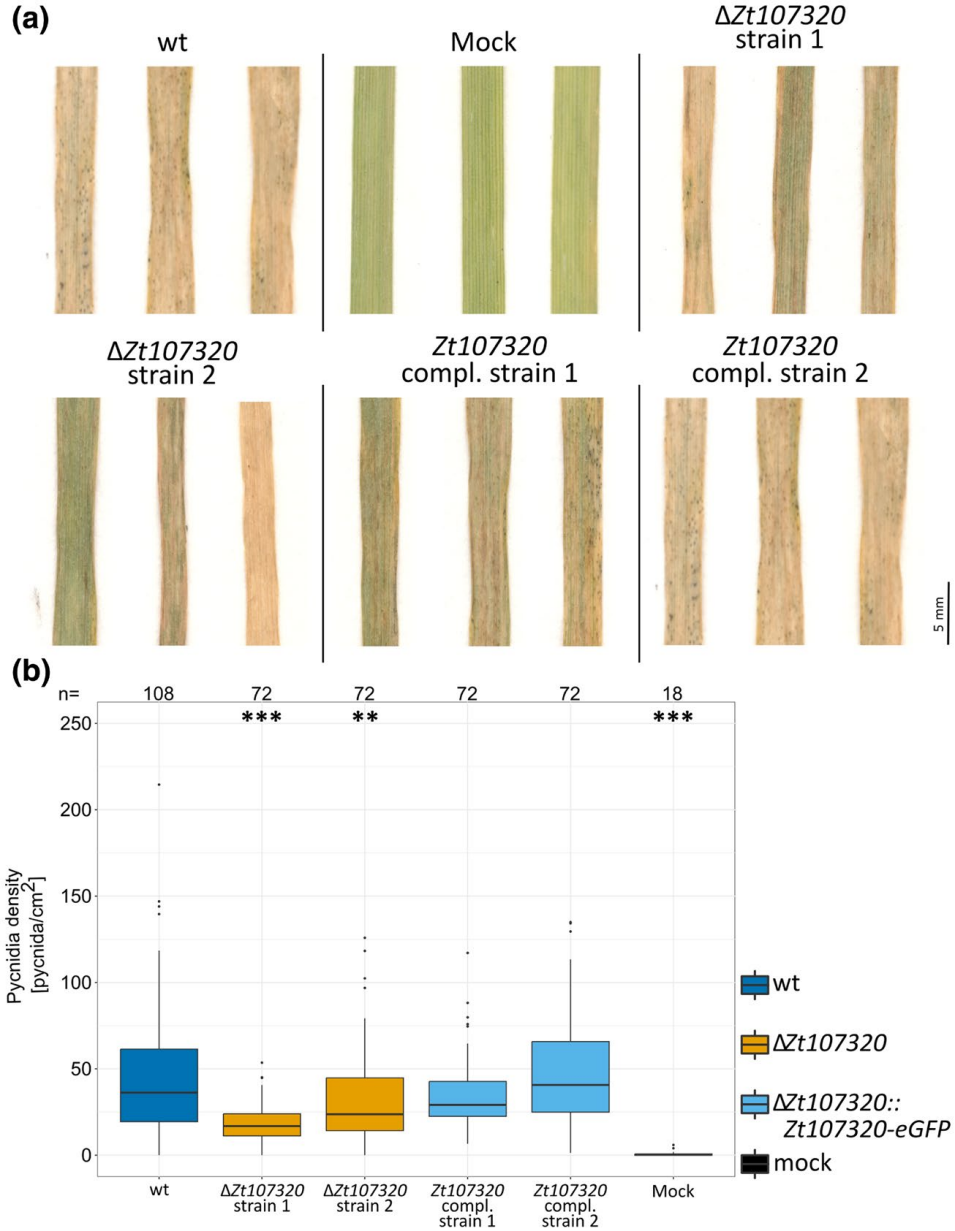
*Zt107320* deletion affects pycnidia production during a compatible infection of wheat. (a) Pictures of representative leaves infected with the *Zymoseptoria tritici* wild type (wt), two independent deletion strains (∆*Zt107320*), two independent complementation strains (∆*Zt107320*::*Zt107320*‐*eGFP*) and mock‐treated leaves. (b) Boxplot depicting the pycnidia density (pycnidia per cm^2^ leaf) pooled from two independent experiments. Number of leaves (*n*) for each strain is indicated along the top. Statistical significance, inferred through an ANOVA and subsequent post hoc Tukey’s HSD comparing the deletion and complementation strains to the wild type, is indicated as *P < 0.05, **P < 0.005, ***P < 0.0005.

We next addressed whether the impact of *Zt107320* deletion on growth rate, the dimorphic switch and cell wall properties also influences the ability of *Z. tritici* to infect its host, *T. aestivum*. We inoculated a predefined area of the second leaf of the susceptible wheat cultivar Obelisk and measured the number of pycnidia at 21 dpi. We observed a pronounced reduction in the production of pycnidia for the *Zt107320* deletion strains. The density of pycnidia per cm^2^ of infected leaf area was reduced significantly for both independent deletions of *Zt107320* (ANOVA, *P* < 1 × 10^−7^, *P* = 0.002) (Fig. 6a,b) compared to the wild type. Complementing the *Zt107320* *in locus* fully restored the wild type phenotype *in planta*. The median for density of pycnidia per cm^2^ of leaf was reduced to 55% (range 30–65%) for the two independent deletion strains and restored to 95% (range 58–116%) for the two independent complementation strains. No significant effect was detected on the ability of the wild type, the deletion and complementation strains to induce necrosis. As the density of pycnidia is considered to be a quantitative measure for virulence (Stewart *et al.*, 2016), significantly decreased pycnidia production indicates reduced virulence of mutants. Thus, we conclude that the putative transcription factor Zt107320, by its effect on the growth rate, the dimorphic switch and the cell wall properties, affects the fitness of *Z. tritici* during infection of the compatible wheat host.

## Discussion

We show that the putative transcription factor Zt107320, belonging to the fungal Zn(II)_2_Cys_6_ family, is involved in the infection programme of *Z. tritici* during a compatible interaction with the host plant *T. aestivum*. Transcription of *Zt107320* is specifically induced during infection of wheat but not during the infection of the non‐host *B. distachyon*. This suggests a functional role of this gene in the regulation of the infection programme during a compatible host–pathogen interaction that allows the fungus to overcome host defences and propagate in the mesophyll tissue.

To date only a small number of genes have been shown to be involved in the virulence of this important wheat pathogen (Motteram *et al.*, 2011; Orton *et al.*, 2011; Poppe *et al.*, 2015; Rudd, 2015; Yemelin *et al.*, 2017). These genes encode the chitin‐binding LysM effector Mg3LysM (Lee *et al.*, 2014; Marshall *et al.*, 2011), two transcription factors ZtWor1 and ZtVf1 (Mirzadi Gohari *et al.*, 2014; Mohammadi *et al.*, 2017), three rapidly evolving small proteins (Hartmann *et al.*, 2017; Poppe *et al.*, 2015; Zhong *et al.*, 2017) and members of more general processes in metabolism and cell signalling [reviewed in Rudd (2015)]. Indeed, in several studies multiple effector candidate genes have been deleted but for most mutants no or small effects on pathogenicity were observed (Gohari *et al.*, 2015; Rudd *et al.*, 2015). Therefore, a high level of functional redundancy seems to be present in *Z. tritici*. The deletion of *Zt107320* results in a relatively small but significant reduction (by approximately 45%) in the pycnidia density, which contrasts to the fully or partially avirulent phenotype demonstrated for the two previously deleted transcription factors ZtWor1 and ZtVf1, respectively (Mirzadi Gohari *et al.*, 2014; Mohammadi *et al.*, 2017). Similar to our observation in the *Zt107320* deletion mutant, deletion of *MoCOD1*, the rice blast homologue of *Zt107320*, led to a quantitative reduction in lesion size and number (Chung *et al.*, 2013). This partial, but incomplete, quantitative reduction in virulence of ∆*Zt107320* strains corresponds with the reduced, but still substantial, growth rate of these deletion mutants *in vitro*. Therefore, other mechanisms independent of Zt107320 are involved in the regulation of growth and development *in vitro* and *in planta*. Similarly, a partial, but incomplete, reduction of invasive growth *in planta* is observed in ∆*MoCOD1* (Chung *et al.*, 2013). The impact of Zt107320 on fungal growth and pathogenicity is similar to the effect caused by other members of the Zn(II)_2_Cys_6_ transcription factor family: TPC1 regulates invasive, polarized growth and virulence in the rice blast fungus *M. oryzae* (Galhano *et al.*, 2017), and the transcription factor FOW2 is known to control the ability of *F. oxysporum* to invade roots and colonize plant tissue (Imazaki *et al.*, 2007).

Zt107320 appears to be involved in the morphological switch from yeast‐like to hyphal growth as deletion of *Zt107320* led to an increase in hyphal growth. More hyphal growth can be observed on MM and MM containing different carbon sources for the *Zt107320* deletion strains compared to the wild type (Figs 5, S3 and S4). In particular the deletion mutants appear to not only grow more hyphae but also branch more from hyphae. The switch to filamentous growth is considered to be essential for plant infection (Kema *et al*., 1996b; Yemelin *et al.*, 2017). For several factors that affect the dimorphic switch, an effect on the pathogenicity has been described. One of the first identified factors that regulates the dimorphic switch in *Z. tritici* was ZtHog1 (synonym: MgHog1), which is required for the dimorphic switch. Deleting *ZtHog1* results in failure to infect wheat leaves (Mehrabi *et al.*, 2006). Glycosylation also affects the switch to hyphal growth. Deletion of *ZtAlg2*, which encodes a putative mannosyltransferase in *Z. tritici*, prevents this switch and results in nonpathogenic strains (Motteram *et al.*, 2011). In addition, the deletion of a putative glycosyltransferase, ZtGt2, was shown to reduce the ability to extend hyphae on solid surfaces and pathogenicity (King *et al.*, 2017). Similarly, the deletion of *MCC1,* which encodes a putative c‐type cyclin, reduces the ability to produce aerial mycelia and impairs pathogenicity (Choi and Goodwin, 2011). This frequently described correlation between the dimorphic switch and pathogenicity was used as means to identify factors involved in pathogenicity in studies that screened for mutants with reduced hyphal growth. Many of these mutants were indeed also reduced in pathogenicity (Yemelin *et al.*, 2017). Therefore, the involvement of Zt107320 in regulating this dimorphism underscores its importance for pathogenicity. Interestingly, in contrast to all earlier reports linking the dimorphic switch and pathogenicity in *Z. tritici*, the deletion of *Zt107320* increases hyphal growth but decreases pathogenicity. This might be due to the effect of Zt107320 on the carrying capacity *k* in MM. The reduction of the carrying capacity in the *Zt10732*0 deletion mutants, but not on the growth rate *r*, may indicate an effect of the transcription factor on carbon utilization once the carbon source becomes limited when approaching stationary phase. The differences in carrying capacity for the ∆*Zt107320* strains could be indicative of an earlier nutrient limitation due to lower efficiency in nutrient usage. This nutrient limitation could therefore be responsible for the more pronounced switch to hyphal growth, in particular, since carbon limitation increases mycelial growth (Francisco *et al.*, 2019). This is further supported by the fact that 24 h after inoculation onto solid MM no differences in growth pattern were observable between the wild type, the *Zt107320* deletion and complementation strains (data not shown). At this time point nutrients could be considered to be non‐limiting. It is therefore interesting to speculate that the effect of the deletion of *Zt107320* on the pathogenicity of *Z. tritici* could be due to the less efficient nutrient utilization during the course of the plant infection resulting in reduced pycnidia density.

Based on RT‐qPCR data we observed that *Zt107320* is differentially expressed between non‐host and host infections and is further highly expressed during infections of compatible hosts. Although previous RNA‐Seq‐based transcriptome studies found *Zt107320* to be expressed at different relative levels compared to liquid culture, both studies showed that it is up‐regulated during later stages of wheat infection and associated with necrotrophic host colonization, indicating a possible function for pycnidia formation (Haueisen *et al.*, 2019; Rudd *et al.*, 2015). Together, these findings support a role of Zt107320 in regulation of growth and pathogenicity of *Z. tritici*.

Interestingly, xylose, the main product of hemicellulose degradation by fungal xylanases, had a pronounced effect on the growth pattern of *Z.* *ritici*. Xylose supplementation not only increased overall growth compared to pure minimal medium but also led to an increase in hyphal growth. In *Z. tritici*, the switch to necrotrophic growth after an initial phase of symptomless infection, overall disease severity and quantitative pycnidiospore production are associated with the activity of endo‐β‐1,4‐xylanase (Siah *et al.*, 2010; Somai‐Jemmali *et al.*, 2017). During the switch to necrotrophy *Z. tritici* rapidly develops large hyphal networks and uses plant‐derived nutrients (Haueisen *et al.*, 2019; Rudd *et al.*, 2015). The observed effect of xylose on the growth morphology suggests that xylose—next to its role as a carbon source—may also direct growth and promote the spatial expansion of the intrafoliar hyphal network during the fungal lifestyle switch to necrotrophic growth. Indeed, genes encoding xylanases were shown to evolve under positive selection (Brunner *et al.*, 2013), indicating an important functional role of this class of enzymes for adaptation to wheat infection. Although functional analyses of several xylanases in other plant pathogenic fungi resulted in no direct phenotypic effect (Brunner *et al.*, 2013; Douaiher *et al.*, 2007), xylanases have been proposed as virulence factors in *Z. tritici* (Douaiher *et al.*, 2007). The results presented here indicate a possible role of xylose as a host infection‐associated signal molecule for *Z. tritici* and should warrant further analysis.

It is important to note that on resequencing of the wild type IPO323 isolate used in this study, we detected a 405 bp in‐frame deletion in the first exon of the *Z. tritici* homologue of white collar 1 (*wc‐1,* gene_ID: Zt09_chr_11_00064, Grandaubert *et al*. 2015) compared to the IPO323 reference genome. Such spontaneous genetic changes were previously reported to occur at an elevated frequency in *Z. tritici* during *in vitro* propagation (Möller *et al.*, 2018). WC‐1 together with WC‐2 composes the White Collar complex (WCC) and acts as a transcription factor whose activity is regulated by light (Corrochano, 2019; He *et al*. 2002; Linden and Macino, 1997). In *Neurospora crassa,* the WCC was shown to affect the transcription of 5.6% of all expressed genes (Chen *et al.*, 2009). It is therefore conceivable that the *wc‐1* partial deletion has pleiotropic effects and may be responsible for the lower pathogenicity of the IPO323 isolate used in this study as compared to work published by other research groups. This would be in line with the observation that in *M. oryzae* the WC*‐*1 orthologue is involved in the regulation of virulence traits (Kim *et al.*, 2011). Because WC‐1 is an important transcriptional regulator that can affect virulence, it is conceivable that the effects we observed for the putative transcription factor Zt107320 were influenced by epistatic effects between *wc‐1* and *Zt107320.* In particular, the regulatory networks that comprise Zt107320 and WC‐1 might overlap or interfere with each other. Therefore, although all strains in this study had the *wc‐1* partial deletion, the ∆*Zt107320* phenotypes could have been affected by the *wc‐1* partial deletion in a non‐additive manner (i.e. positive/negative epistasis). As a consequence, the phenotype of ∆*Zt107320* in a strain that does not have the *wc‐1* partial deletion may differ from the phenotype that we describe here. Nevertheless, with the complementation test included in our study, we can conclude that *Zt107320* influences the dimorphic switch, growth and virulence of *Z. tritici*. Future studies should address the role WC‐1 might play in the regulation of virulence in *Z. tritici* and the potential interaction between Zt107320 and WC‐1.

In conclusion, we showed that Zt107320 affects the fitness of the wheat pathogen *Z. tritici*. *Zt107320* is differentially expressed in host and non‐host environments, and is up‐regulated during the early stages of infection on compatible hosts and down‐regulated on non‐hosts. This down‐regulation corresponds to a considerably reduced growth and halted infections of *Z. tritici* after stomatal penetration of the non‐host *B. distachyon*. In addition, we can confirm that Zt107320 has a nuclear localization, consistent with its putative function as a transcription factor, and that it further regulates the dimorphic switch between yeast‐like and hyphal growth that is considered to be essential for pathogenicity. We therefore hypothesize that the putative transcription factor Zt107320 is part of the regulatory network that controls growth and development, and could be involved in integrating signals that differ between compatible and non‐compatible infections. Future studies should address the specific target genes of Zt107320 and their expression pattern during compatible and non‐compatible interactions. Furthermore, the transcriptional regulation of *Zt107320* suggests that specific signals in the compatible host–pathogen interaction in wheat are responsible for the up‐regulation of this particular transcription factor‐encoding gene. Identification of these host‐derived signals will provide a fundamental insight into the molecular basis of host–pathogen interaction and host specificity in *Z. tritici*.

## Experimental Procedures

### Fungal strains and plant material

The Dutch isolate IPO323 was kindly provided by Gert Kema (Wageningen, Netherlands) and is available from the Westerdijk Institute (Utrecht, Netherlands) with the accession number CBS 115943. The isolate used in our experiments lacked accessory chromosome 18, presumably lost during culture maintenance *in vitro* (Kellner *et al.*, 2014), and contains a 405 bp in‐frame deletion in gene Zt09_chr_11_00064 (chr 11:223,109‐223,513), a homologue of the white collar 1 (*wc‐1*) gene involved in controlling the expression of light‐regulated genes in *N. crassa* (Linden and Macino, 1997). Strains were maintained in either liquid yeast malt sucrose (YMS) broth (4 g/L yeast extract, 4 g/L malt extract, 4 g/L sucrose) at 18 °C on an orbital shaker or on solid YMS (+20 g/L agar) at 18 °C. The *T. aestivum* cultivar Obelisk was obtained from Wiersum Plantbreeding BV (Winschoten, Netherlands). *Brachypodium distachyon* inbred line Bd21 was kindly provided by Thierry Marcel (Bioger, INRA, France).

### Sequence analysis

Phylogenetic analysis of Zt107320 was conducted using the software Geneious Prime v. 2019.0.4 (https://www.geneious.com). Protein sequences with homology to the translated protein sequence of *Zt107320* were retrieved from the Ensemble Fungi database (Kersey *et al.*, 2018). Furthermore, we included Zt107320 and its homologues from the sister species *Z. pseudotritici* and *Z. ardabiliae*, and the non‐orthologous sequences of the well‐described transcription factor AmyR from *A. niger* and MalR from *A. oryzae*, and the closely related gene MYCGRDRAFT_111096 from *Z. tritici.* A total of 30 matches were retrieved from the Ensemble Fungi database. Alignments were constructed using MUSCLE (Edgar, 2004) and trees constructed using the neighbour‐joining algorithm, building a consensus tree with 1000 bootstrapping replicates. Protein domains of the consensus sequence were identified using InterProScan (Quevillon *et al.*, 2005). Prediction of the nuclear localization of the Zt107320 protein was conducted using the WoLF PSORT predictor (Horton *et al.*, 2007).

### Analysis of *Z. tritici* during its compatible and non‐compatible infections by confocal microscopy

Morphology and development of *Z. tritici* within and on the surface of leaves of *B. distachyon* inbred line Bd21 were analysed by confocal laser‐scanning microscopy (CLSM) as described previously (Haueisen *et al.*, 2019). Analyses of compatible *Z. tritici* infections on *T. aestivum* 'Obelisk' were conducted by combining microtomy and CLSM as previously described (Rath *et al.*, 2014). Distinct areas of the second leaf of 12‐day‐old (Bd21) and 14‐day‐old (wheat) seedlings were brush‐inoculated with 1 × 10^7^ cells/mL in 0.1% Tween 20. Plants were incubated at 22 °C (day)/ 20 °C (night) and 100% humidity with a 16‐h light period for 48 h. Subsequently, humidity was reduced to 70%. Microscopy was conducted using a Leica TCS SP5 and analysis of imaging of *z*‐stacks was done using Leica Application Suite Advanced Fluorescence (Leica Microsystems, Germany) and AMIRA (FEITM Visualization Science Group, Germany).

### Analysis of Zt107320‐eGFP expression using confocal microscopy

Cells were grown on solid YMS medium for 7 days before being scraped off the medium surface and introduced into 10 mM phosphate buffer (pH 7.2) containing 1 µg/µL Hoechst 33342 (Sigma‐Aldrich Chemie GmbH, Munich, Germany). Cells were incubated for 15–30 min in the dark and then transferred to a microscope slide and analysed by CLSM.

### RNA isolation and RT‐qPCR

We analysed gene expression patterns of *Zt107320* of *Z. tritici* using a quantitative reverse transcription PCR (RT‐qPCR) experiment. Total RNA was extracted from fungal axenic cultures (grown for 72 h in YMS medium at 18 °C and 200 rpm) and from snap‐frozen leaf tissue infected with *Z. tritici* (4, 8, 14 and 21 dpi) using the TRIzol reagent (Invitrogen, Karlsruhe, Germany), following the manufacturer’s instructions. Three biological replicates were included in the experimental set‐up. The cDNA samples were used in a RT‐qPCR experiment employing the iQ SYBR Green Supermix Kit (Bio‐Rad, Munich, Germany). PCR was conducted in a CFX96 RT‐PCR Detection System (Bio‐Rad) with the constitutively expressed control gene glyceraldehyde‐3‐phosphate dehydrogenase (GAPDH). All primers are listed in Table S1.

### Generation of *Zt107320* deletion and complementation mutants by gene replacement

Fungal transformations and the creation of deletion and complementation mutants of *Zt107320* were conducted as previously described (Poppe *et al.*, 2015). In brief, gene deletions were generated by amplifying an approximately 1 kb region of the 5’ and 3’ flanking regions of *Zt107320* using PCR. The amplified flanking sequences were fused to a hygromycin resistance (*H* *ygR*) cassette and an *Eco*RV cut vector backbone (pES61) using Gibson assembly (Gibson *et al.*, 2009). Electrocompetent cells of the *Agrobacterium tumefaciens* strain AGL1 were transformed using standard protocols. These transformed *A. tumefaciens* cells were used for the transformation of *Z. tritici* as previously described (Zwiers *et al*., 2001). The same strategy was applied for the generation of the complementation strains by a C‐terminal fusion of the *Zt107320* gene with an eGFP tag and a geneticin resistance cassette (*NeoR*) as a selection marker (Fig. S5). After transformation and homologous recombination in the ∆*Zt107320* strain 1, the hygromycin resistance cassette was replaced by *Zt107320‐eGFP* and the geneticin resistance cassette (Fig. S5). Homologous recombination and integration were confirmed using PCR and Southern blot analysis by standard protocols. In short, genomic DNA was isolated using phenol‐chloroform isolation (Sambrook and Russell, 2001). Restriction digestion was performed using *Pvu*II, followed by gel electrophoresis, blotting and detection using digoxygenin‐labelled probes binding to the upstream and downstream flank of *Zt107320* (Fig. S5). In total four ∆*Zt107320* strains and two Δ*Zt107320*::*Zt107320*‐eGFP strains were generated.

### *In vitro* phenotyping

The *Z. tritici* strains were grown on YMS solid medium for 5–7 days at 18 °C before the cells were scraped off the plate surface. For the determination of the growth rates, the cells were resuspended and counted. The cell density was adjusted to 50 000 cells/mL and 175 µL of the cell suspension was added to a well of a 96‐well plate. Plates were inoculated at 18 °C at 200 rpm with the OD_600_ being measured twice daily on a Multiskan Go plate reader (Thermo Scientific, Dreieich, Germany) (Table S2). Estimation of growth rates was done by employing the logistic growth equation as implemented in the growthcurver package v. 0.2.1 in R v. R3.4.1 (R Core Team, 2015). For the determination of the *in vitro* phenotypes, the cell number was adjusted to 10^7^ cells/mL in ddH_2_O and serially diluted to 10^3^ cells/mL. Three microlitres of each cell dilution was transferred onto YMS agar containing the tested compounds and incubated for 7 days at 18 or 28 °C. To test high osmotic stresses, 0.5 M NaCl, 1 M NaCl, 1 M sorbitol, 1.5 M sorbitol and 2 M sorbitol (Carl Roth GmbH, Karlsruhe, Germany) were added to the YMS solid medium. To test cell wall stresses, 300 µg/mL and 500 µg/mL Congo red and 200 µg/mL Calcofluor (Sigma‐Aldrich Chemie GmbH, Munich, Germany) were added to the YMS solid medium. Finally, to determine the effect of reactive oxygen species on mutant growth morphology, 2 mM H_2_O_2_ (Carl Roth GmbH) was added to the YMS solid medium.

To test whether Zt107320 affects the hyphal growth of *Z. tritici* and the ability of the fungus to use different carbon sources, we used MM as described in Barratt *et al.* (1965). Glucose, fructose, xylose, maltose, galactose, sorbitol, mannitol, starch and sucrose were added at a final concentration of 10 g/L and 20 g/L agar was included. Strains were grown on YMS solid medium for 7 days and resuspended into ddH_2_O. Cell number was adjusted and 3 µL of 5 × 10^3^ cells/mL were added to the surface of the MM plates. Plates were incubated at 18 °C in the dark and growth was monitored using an inverse microscope and a stereomicroscope after 24 h and 14 days.

### *In planta* phenotyping

For the *in planta* phenotypic assays, we germinated seeds of the wheat cultivar Obelisk on wet sterile Whatman paper for 4 days before potting using the soil Fruhstorfer Topferde (Hermann Meyer GmbH, Rellingen, Germany). Wheat seedlings were further grown for 7 days before inoculation. *Zymoseptoria tritici* strains were grown on YMS solid medium for 5 days at 18 °C before the cells were scraped from the plate surface. The cell number was adjusted to 10^8^ cells/mL in H_2_O + 0.1% Tween 20, and the cell suspension was brushed onto approximately 5 cm on the abaxial and adaxial sides of the second leaf of each seedling. Inoculated plants were placed in sealed bags containing water for 48 h to facilitate infection through stomata. Plants were grown under constant conditions with a day/night cycle of 16 h light (*c*. 200 µmol/m^2^/s) and 8 h darkness in growth chambers at 20 °C. Plants were grown for 21 dpi at 90% relative humidity. At 21 dpi the infected leaves were cut, taped to sheets of paper and pressed for 5 days at 4 °C before being scanned at a resolution of 2400 dots per inch (dpi) using a flatbed scanner (HP Photosmart C4580, HP, Böblingen, Germany). Scanned images were analysed using an automated image analysis in Image J (Schneider *et al.*, 2012) adapted from Stewart *et al.* (2016). The read‐out pycnidia/cm^2^ leaf surface was used for all subsequent analyses. See Table S3 for a summary of the *in planta* results.

### Statistical analysis

Statistical analyses were conducted in R v. R3.4.1 (R Core Team, 2015) using the suite R Studio v.  1.0.143 (R Studio Team, 2015). Data inspection showed a non‐normal distribution for all datasets, including the measured pycnidia densities (pycnidia/cm^2^). Therefore, we performed an omnibus analysis of variance using rank‐transformation of the data (Conover and Iman, 1981) employing the model: pycnidia density ~ strain × experiment, *r* ~ strain and *k* ~ strain, respectively. Post hoc tests were performed using Tukey's HSD (Tukey, 1949).

## Supporting information


**Fig. S1** Zt107320 impacts maximum carrying capacity *k* but not the growth rate *r* of *Zymoseptoria tritici *in minimal medium containing sucrose and xylose. Maximum growth rate *r* and carrying capacity *k* of fungal cultures grown in liquid minimal medium (MM) containing the indicated carbohydrates as carbon sources. For sucrose and xylose as carbon source a significant effect of the deletion of *Zt107320* on the carrying capacity was discernible, whereas the complementation strains were not statistically significantly different from the wild type (wt). All other carbon sources and the maximum growth rate were not significantly different for the two independent deletion strains. Statistical significance inferred through an ANOVA and subsequent post hoc Tukey’s HSD comparing the deletion and complementation strains to the wild type, is indicated as *P < 0.05; **P < 0.005; ***P < 0.0005.

**Fig. S2** *In vitro* phenotype of *Zt107320* wild type and mutants. *In vitro* growth of the wild type (wt), two independent deletion strains (∆*Zt107320*), two independent complementation strains (∆*Zt107320*::*Zt107320*‐eGFP) on YMS medium including the indicated compounds to assess the effect of osmotic stress (NaCl, sorbitol), reactive oxygen species (H_2_O_2_), cell wall stressors (Calcofluor, Congo Red) and increased temperature (28 °C) on growth and morphology of *Zymoseptoria tritici.*

**Fig. S3** Growth morphologies of* Zymoseptoria tritici *wild type and ∆*Zt107320* and ∆*Zt107320::Zt107320‐eGFP* strains on solid minimal medium in the presence of different carbon sources. Micrographs depicting growth morphologies after 14 days at 18 °C on media. Upper row and lower row: Two independent examples of colony morphology for each condition. Hyphal‐like protrusions originated from primary colonies. Hyphal‐like protrusions appeared more pronounced, more branched and covering a larger area for the two ∆*Zt107320* strains compared to wild type (wt). The two Zt107320::Zt107320‐eGFP strains showed wild type phenotype. Scale bar represents 1 mm.

**Fig. S4** Details of growth morphologies of *Zymoseptoria tritici* wild type and ∆*Zt107320* and ∆*Zt107320::Zt107320*‐*eGFP* strains on solid minimal medium in the presence of different carbon sources. Detailed micrographs depicting growth morphologies after 14 days at 18 °C on media. Upper row and lower row: Details of one hyphal‐like protrusion depicted at differed magnification. Hyphal cells and yeast‐like cells can be seen in all conditions. In the two ∆*Zt107320* strains more hyphal growth and more hyphal branching of hyphae resulting in hyphae occurred in comparison to the wild type (wt). The two ∆*Zt107320::Zt107320‐eGFP* complementation strains showed wild type morphologies. Scale bar represents 50 µm.

**Fig. S5** Generation of *Zt107320* mutants in *Zymoseptoria tritici*. (a) Schematic illustration of the gene replacement strategy used to delete the gene *Zt107320*. Generation of the ∆*Zt107320* mutants by homologous recombination between the upstream (UF) and downstream flanking regions (DF) of *Zt107320 *in its genomic locus and a plasmid carrying the hygromycin resistance cassette (*HygR*) located between the UF and DF. Homologous recombination results in the integration of the hygromycin‐resistance gene cassette (*HygR*) in the locus of *Zt107320*. ∆*Zt107320::Zt107300*‐eGFP were generated by homologous recombination between UF and DF of the ∆*Zt107320 *strains and a transformed plasmid containing a C‐terminal fusion of *Zt107320* and *eGFP* and a geneticin resistance cassette (*NeoR*) located between the UF and DF. Yellow bars indicate the position of the probes used in the Southern blot analyses. (b) Confirmation of *Zt107320* mutants by Southern blot analyses.

**Table S1** List of all primers used within this study.

**Table S2** Summary of *in vitro* growth data.

**Table S3** Summary of *in planta *phenotype.
