# Supplementary figures and images for: The transcription factor Zt107320 affects the dimorphic switch, growth and virulence of the fungal wheat pathogen Zymoseptoria tritici
Source: Mol Plant Pathol. 2019 Nov 8;21(1):124–38. doi: 10.1111/mpp.12886 (PMC6913241; doi:10.1111/mpp.12886)

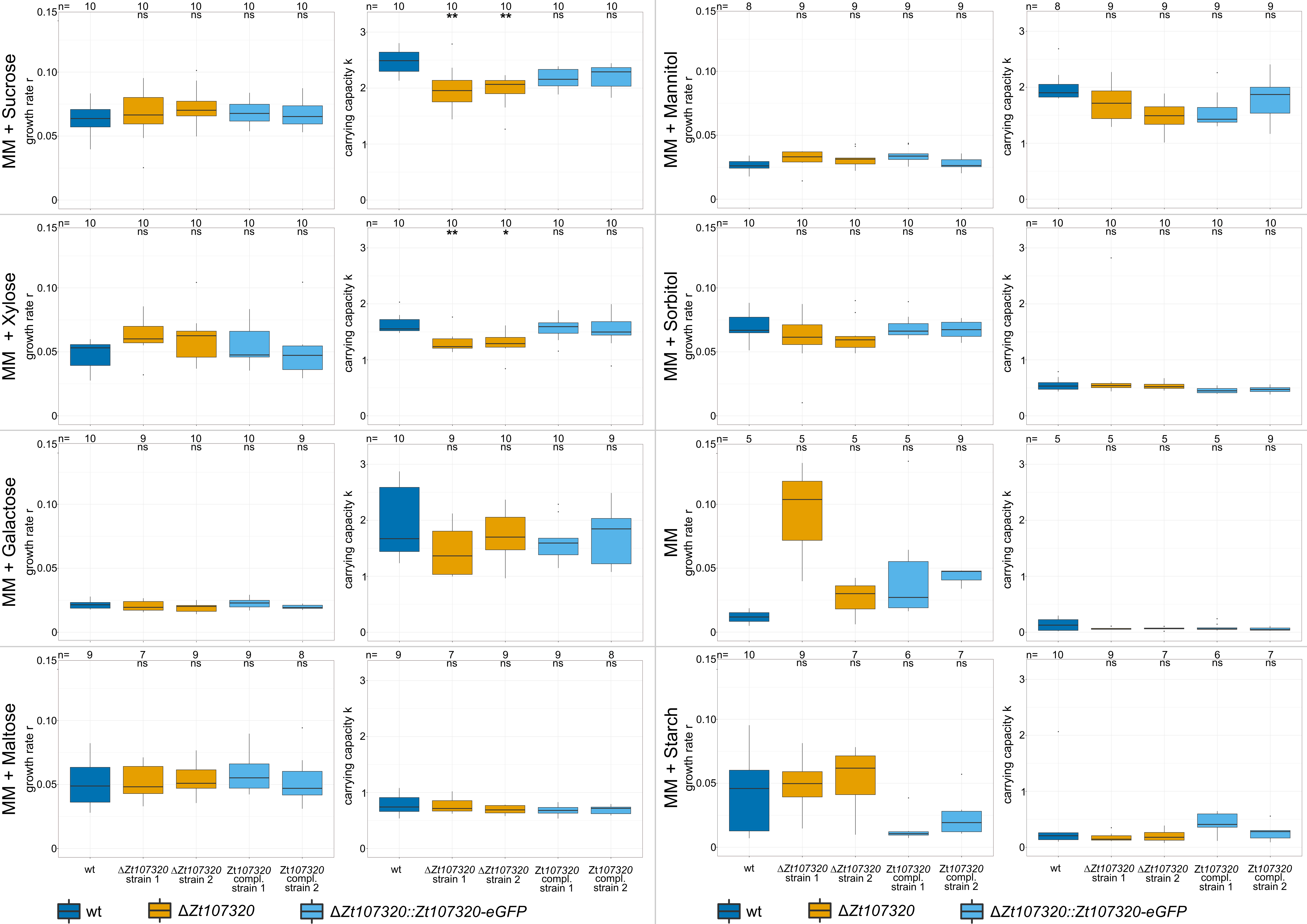

Supplement: Supplementary file 1 — Fig. S1 Zt107320 impacts maximum carrying capacity k but not the growth rate r of Zymoseptoria tritici in minimal medium containing sucrose and xylose. Maximum growth rate r and carrying capacity k of fungal cultures grown in liquid minimal medium (MM) containing the indicated carbohydrates as carbon sources. For sucrose and xylose as carbon source a significant effect of the deletion of Zt107320 on the carrying capacity was discernible, whereas the complementation strains were not statistically significantly different from the wild type (wt). All other carbon sources and the maximum growth rate were not significantly different for the two independent deletion strains. Statistical significance inferred through an ANOVA and subsequent post hoc Tukey’s HSD comparing the deletion and complementation strains to the wild type, is indicated as *P < 0.05; **P < 0.005; ***P < 0.0005. [file MPP-21-124-s001.tiff]

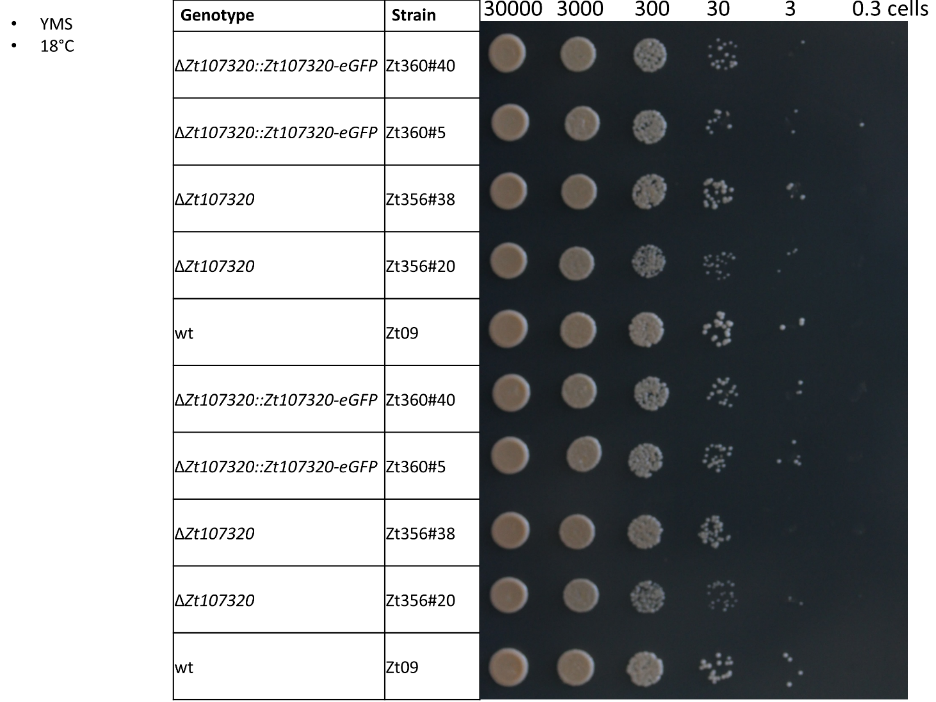


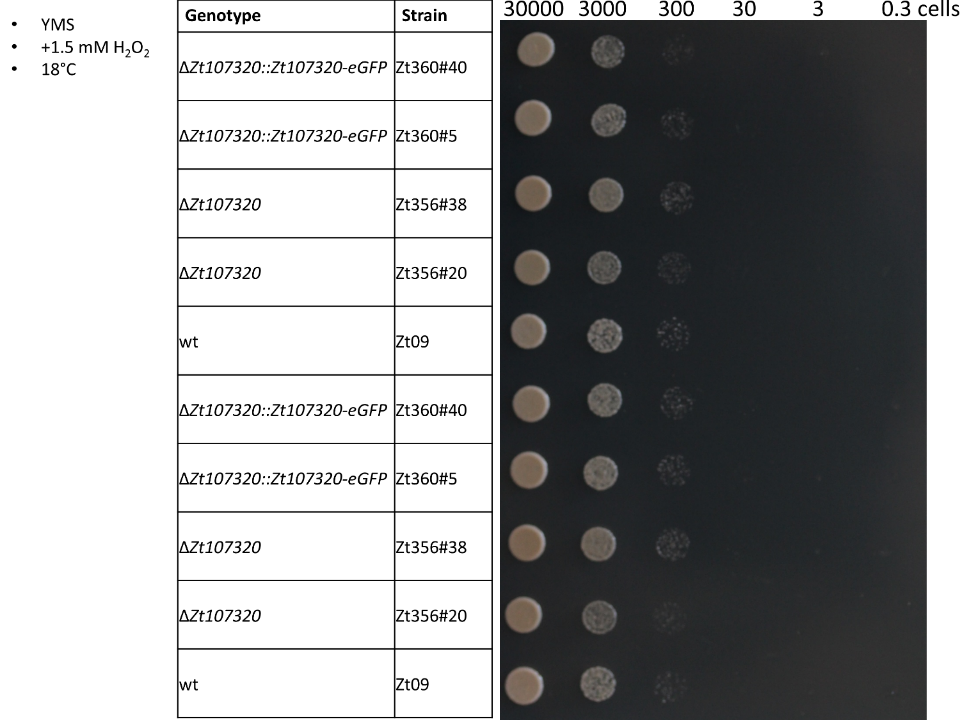


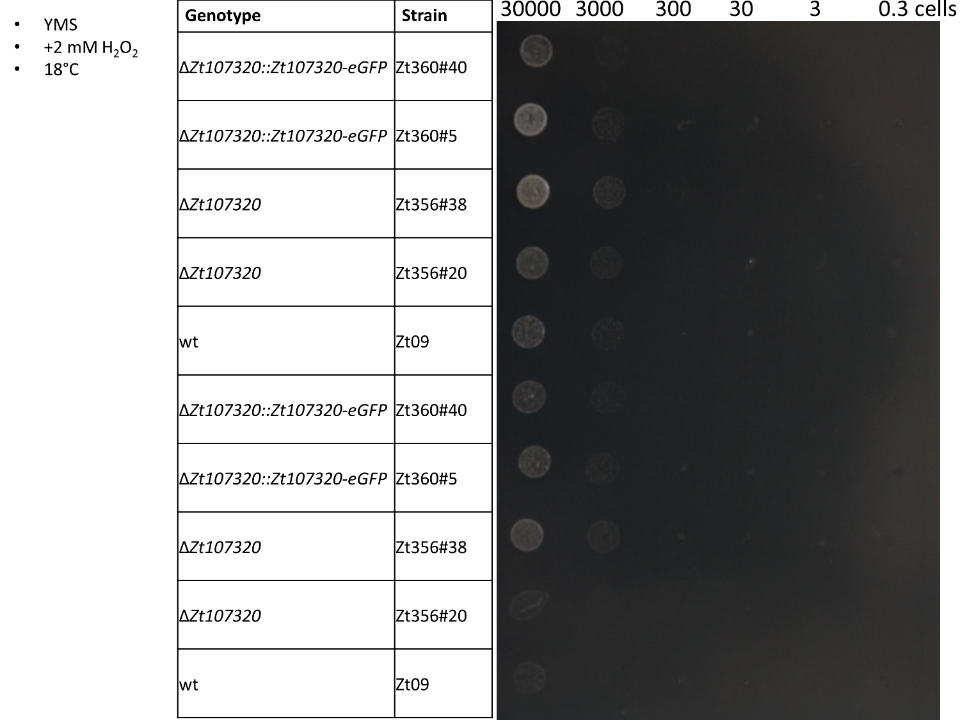


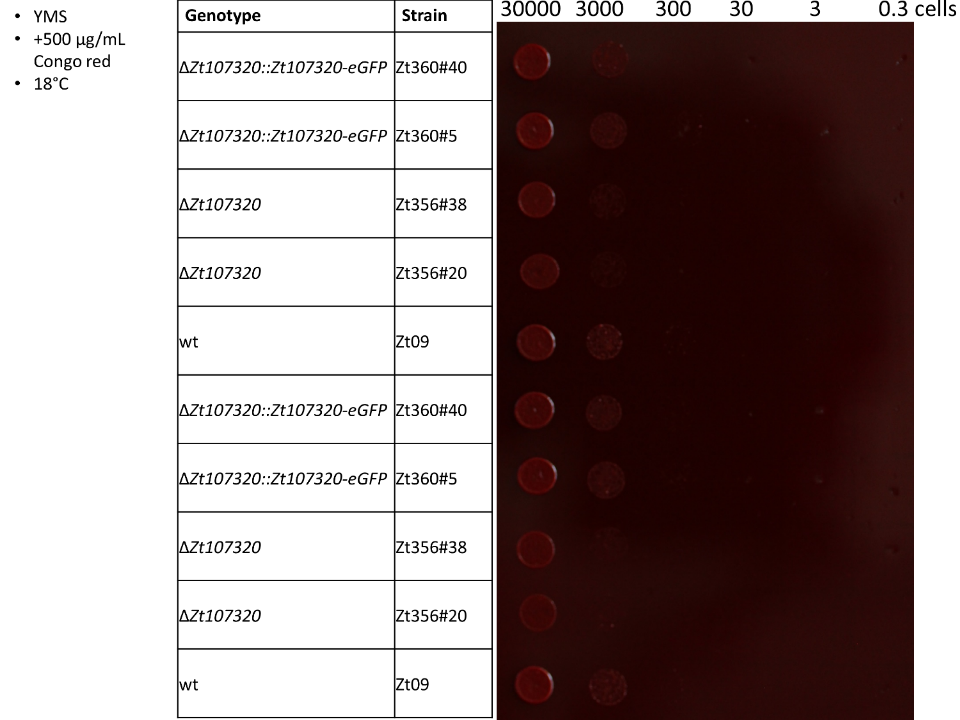


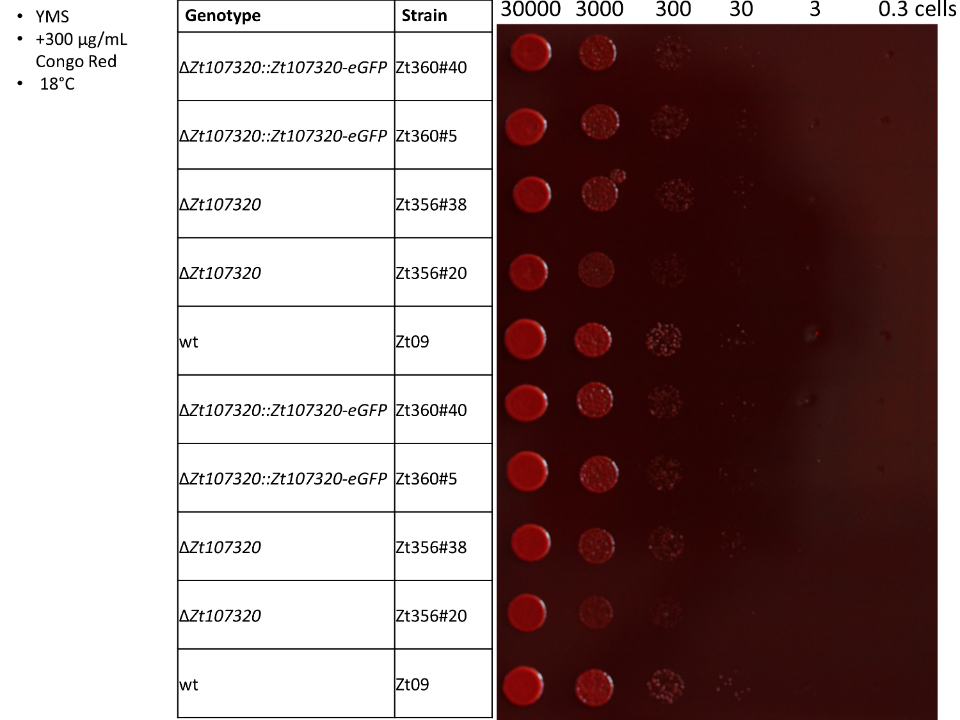


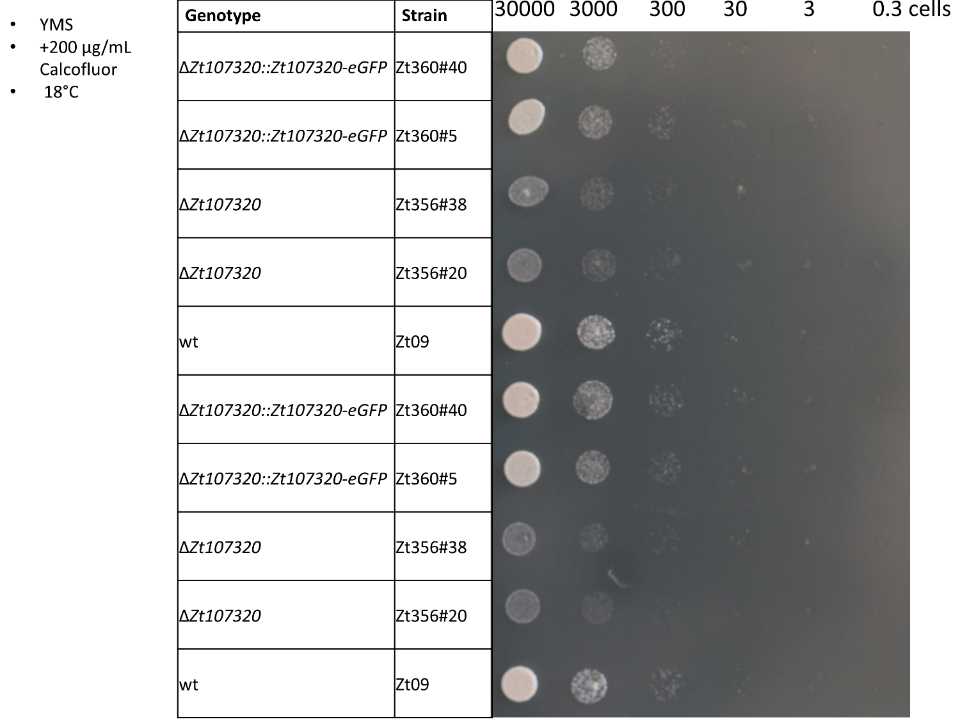


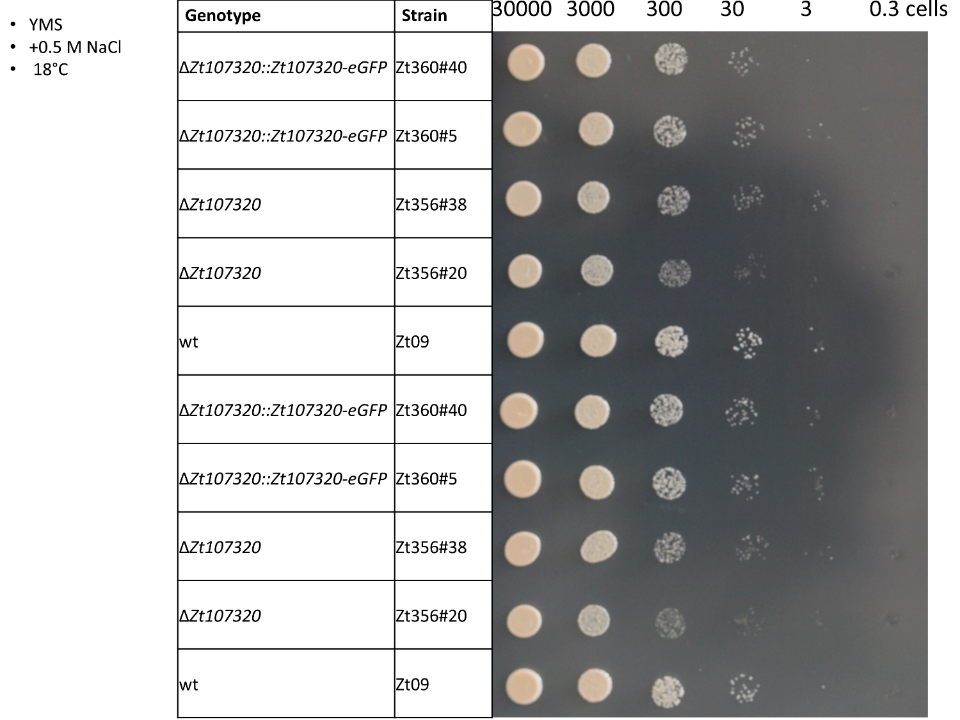


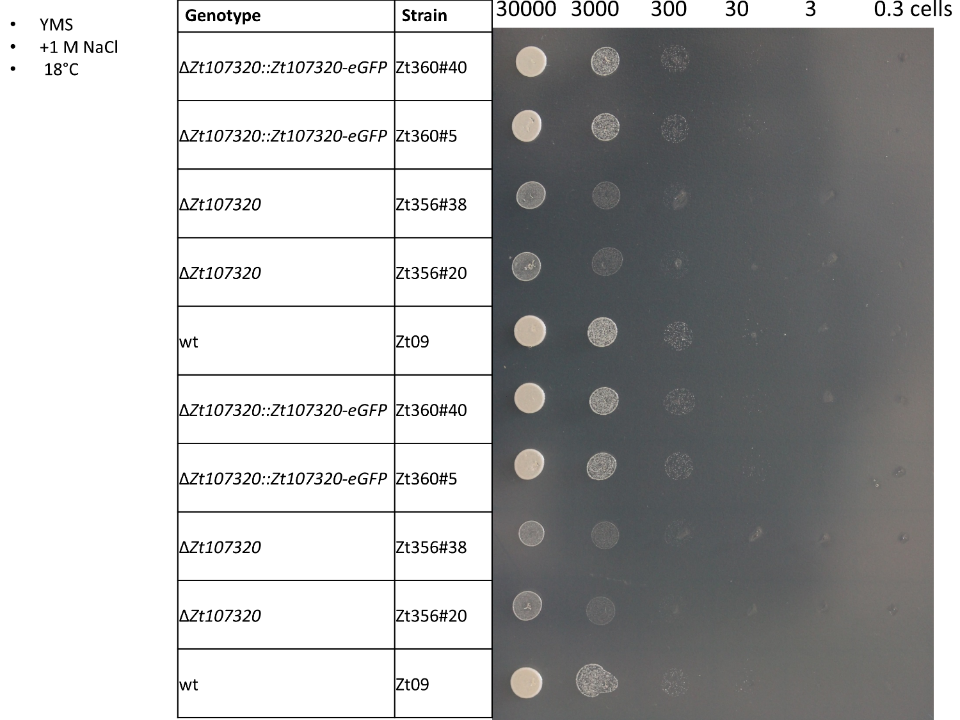


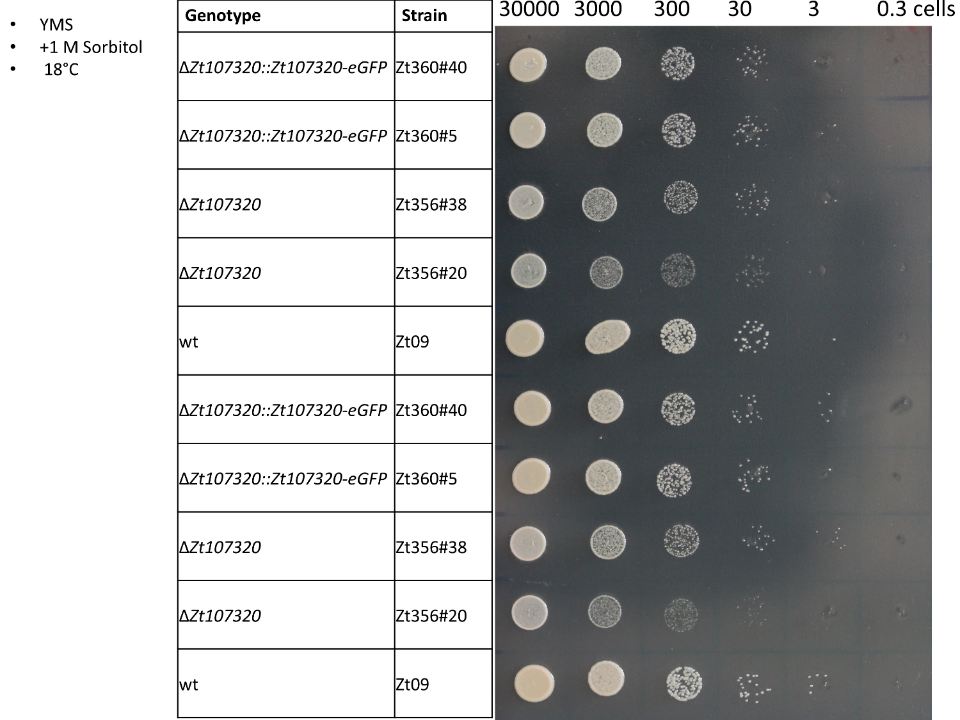


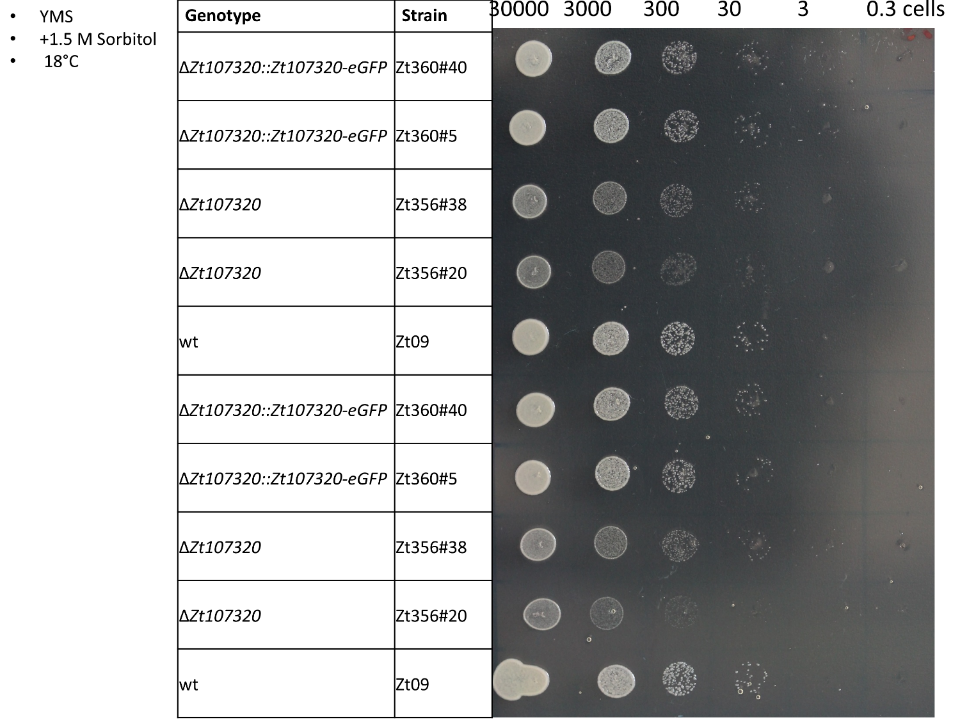


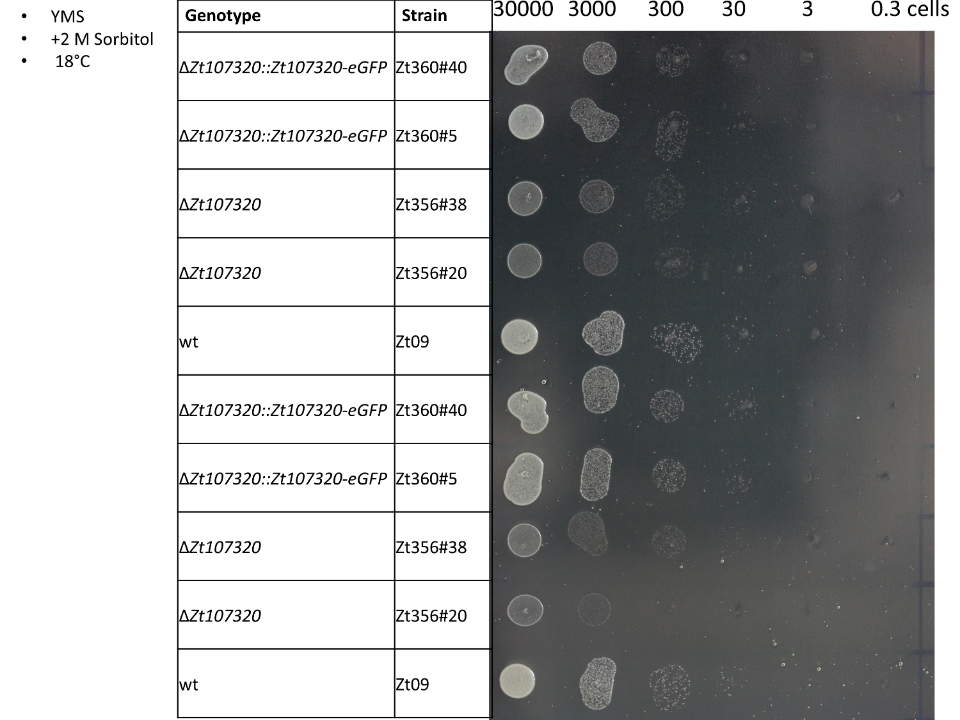


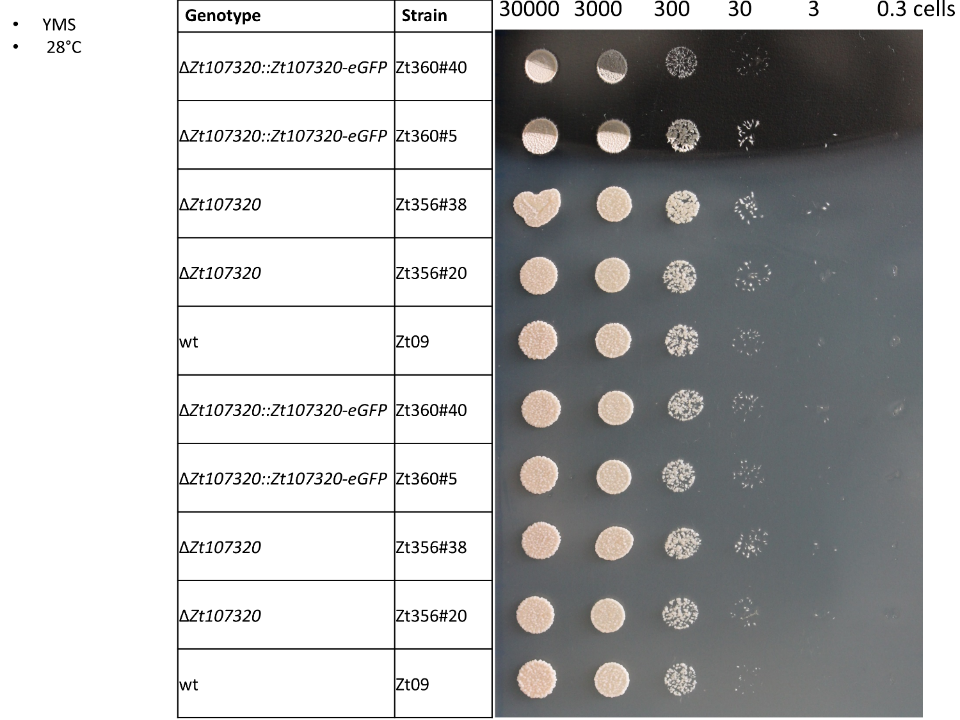

Supplement: Supplementary file 2 — Fig. S2 In vitro phenotype of Zt107320 wild type and mutants. In vitro growth of the wild type (wt), two independent deletion strains (∆Zt107320), two independent complementation strains (∆Zt107320::Zt107320‐eGFP) on YMS medium including the indicated compounds to assess the effect of osmotic stress (NaCl, sorbitol), reactive oxygen species (H2O2), cell wall stressors (Calcofluor, Congo Red) and increased temperature (28 °C) on growth and morphology of Zymoseptoria tritici. [file MPP-21-124-s002.docx]

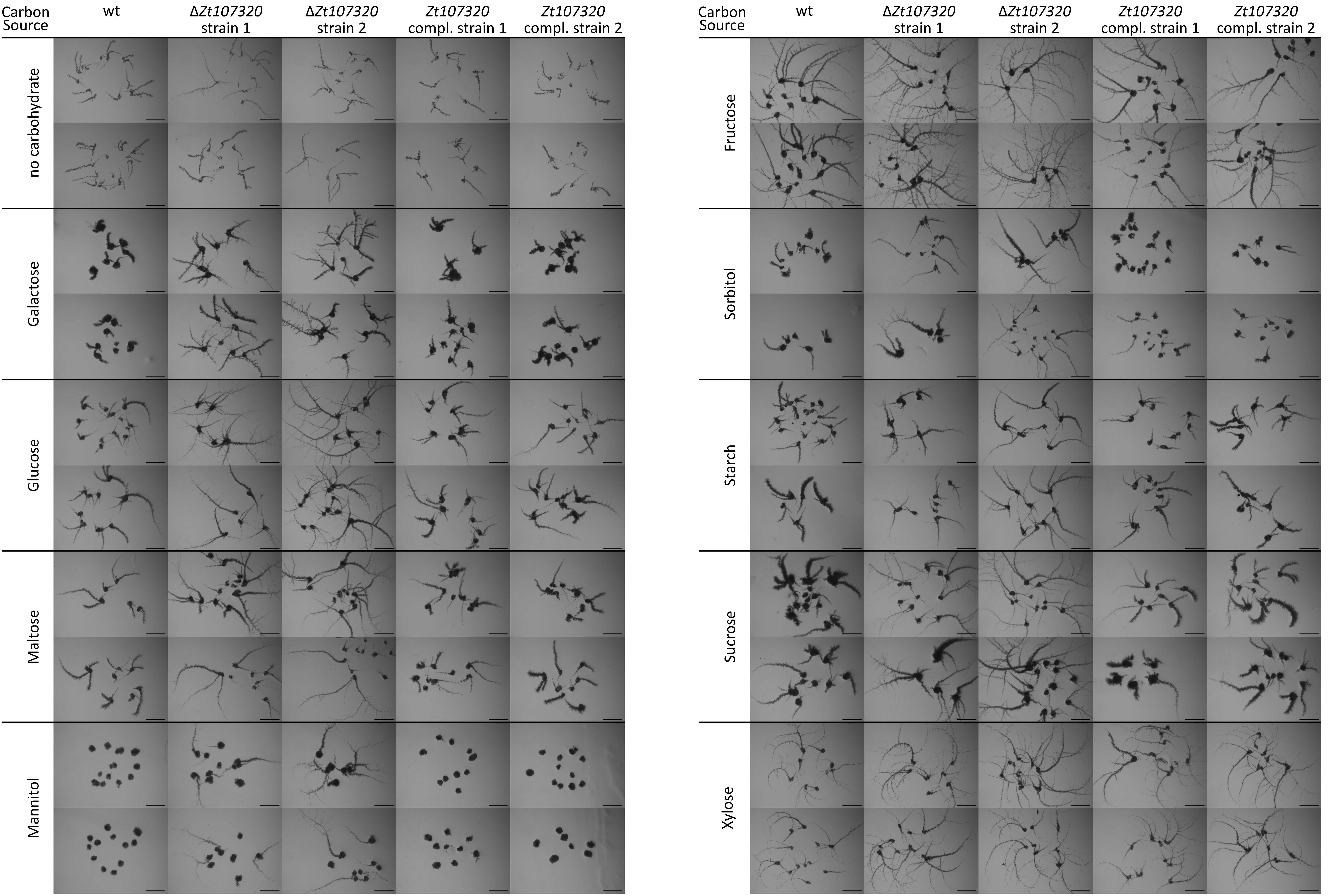

Supplement: Supplementary file 3 — Fig. S3 Growth morphologies of Zymoseptoria tritici wild type and ∆Zt107320 and ∆Zt107320::Zt107320‐eGFP strains on solid minimal medium in the presence of different carbon sources. Micrographs depicting growth morphologies after 14 days at 18 °C on media. Upper row and lower row: Two independent examples of colony morphology for each condition. Hyphal‐like protrusions originated from primary colonies. Hyphal‐like protrusions appeared more pronounced, more branched and covering a larger area for the two ∆Zt107320 strains compared to wild type (wt). The two Zt107320::Zt107320‐eGFP strains showed wild type phenotype. Scale bar represents 1 mm. [file MPP-21-124-s003.tiff]

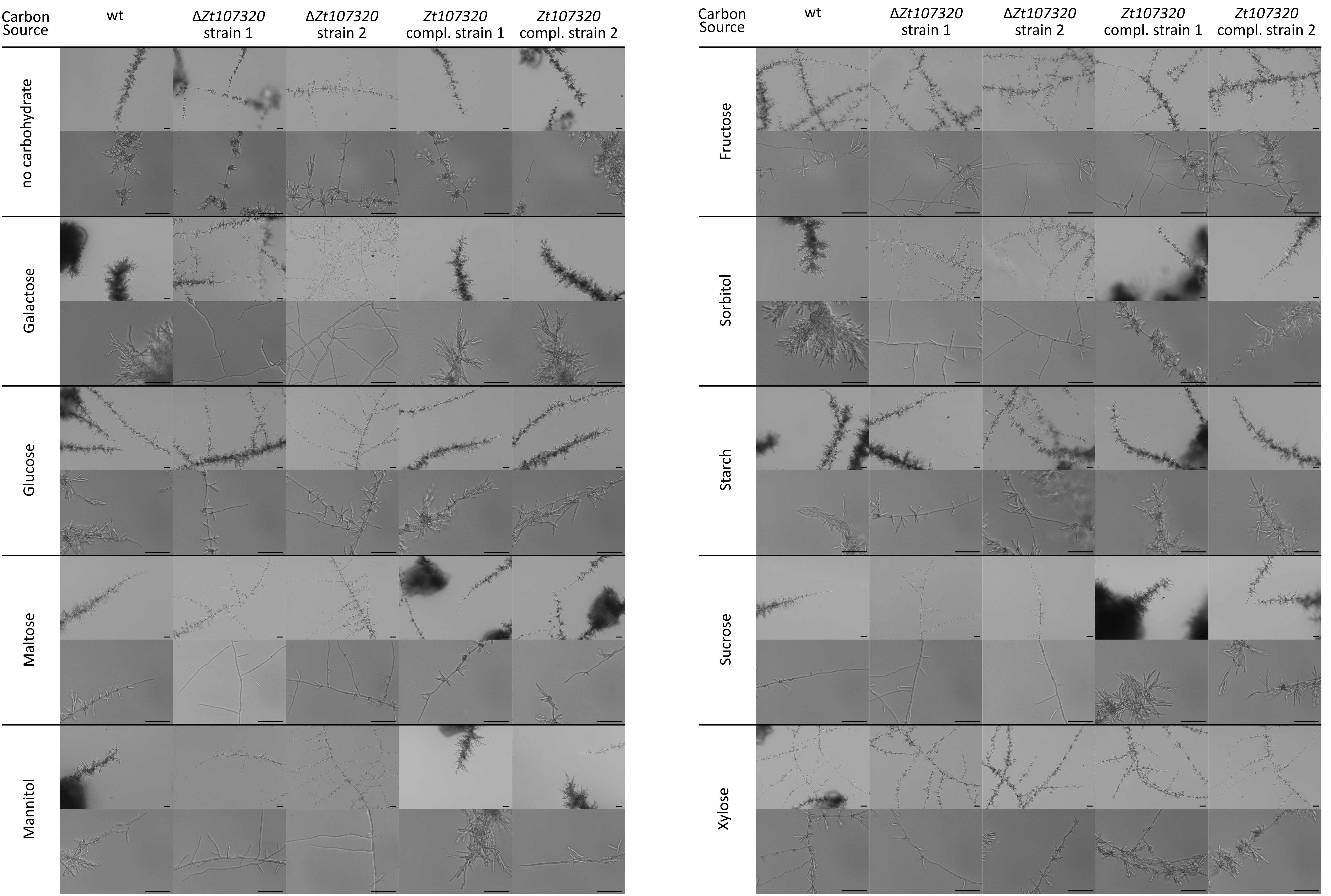

Supplement: Supplementary file 4 — Fig. S4 Details of growth morphologies of Zymoseptoria tritici wild type and ∆Zt107320 and ∆Zt107320::Zt107320‐eGFP strains on solid minimal medium in the presence of different carbon sources. Detailed micrographs depicting growth morphologies after 14 days at 18 °C on media. Upper row and lower row: Details of one hyphal‐like protrusion depicted at differed magnification. Hyphal cells and yeast‐like cells can be seen in all conditions. In the two ∆Zt107320 strains more hyphal growth and more hyphal branching of hyphae resulting in hyphae occurred in comparison to the wild type (wt). The two ∆Zt107320::Zt107320‐eGFP complementation strains showed wild type morphologies. Scale bar represents 50 µm. [file MPP-21-124-s004.tiff]

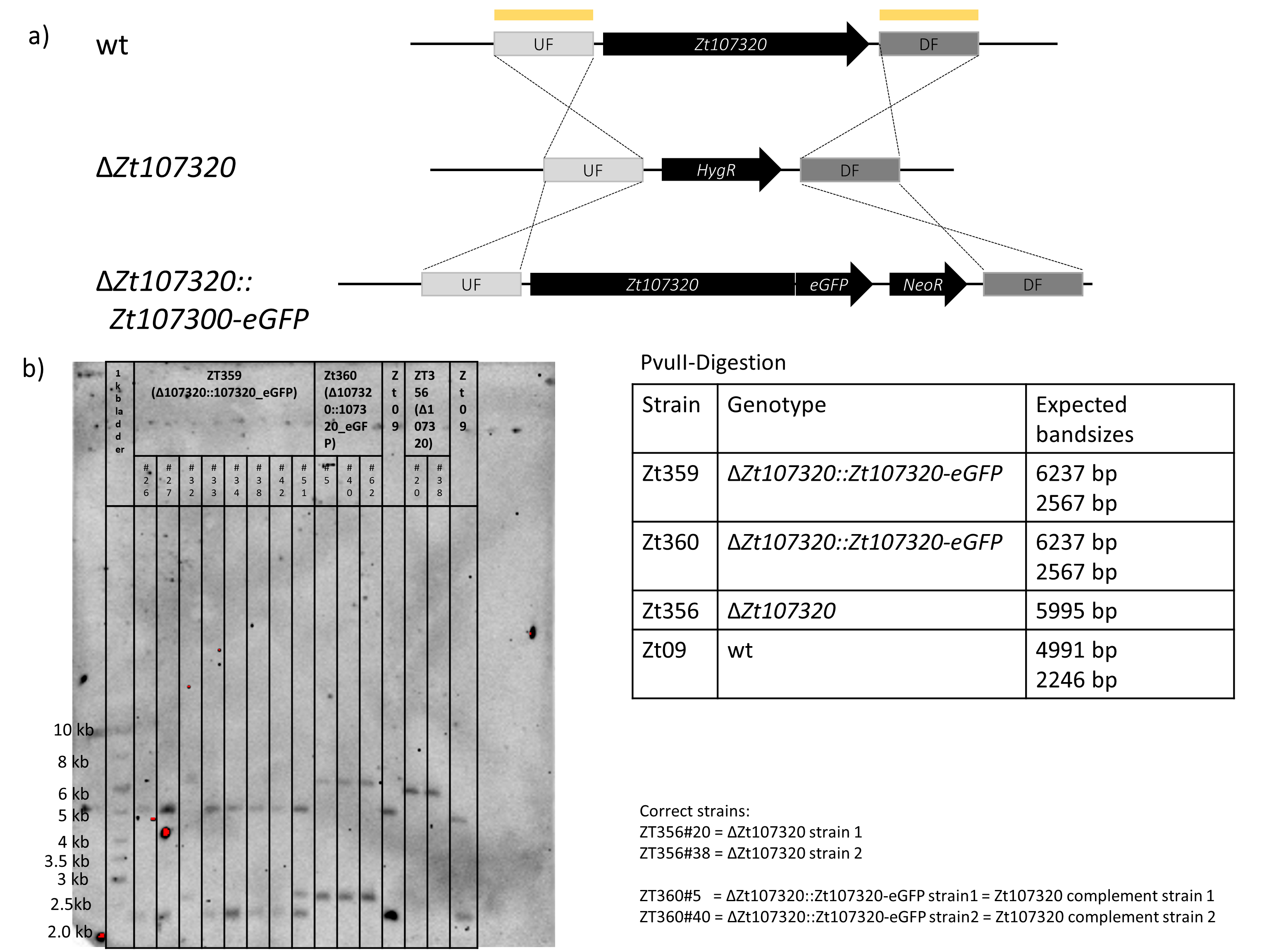

Supplement: Supplementary file 5 — Fig. S5 Generation of Zt107320 mutants in Zymoseptoria tritici. (a) Schematic illustration of the gene replacement strategy used to delete the gene Zt107320. Generation of the ∆Zt107320 mutants by homologous recombination between the upstream (UF) and downstream flanking regions (DF) of Zt107320 in its genomic locus and a plasmid carrying the hygromycin resistance cassette (HygR) located between the UF and DF. Homologous recombination results in the integration of the hygromycin‐resistance gene cassette (HygR) in the locus of Zt107320. ∆Zt107320::Zt107300‐eGFP were generated by homologous recombination between UF and DF of the ∆Zt107320 strains and a transformed plasmid containing a C‐terminal fusion of Zt107320 and eGFP and a geneticin resistance cassette (NeoR) located between the UF and DF. Yellow bars indicate the position of the probes used in the Southern blot analyses. (b) Confirmation of Zt107320 mutants by Southern blot analyses. [file MPP-21-124-s005.tif]
